# Whole blood microRNA expression may not be useful for screening non-small cell lung cancer

**DOI:** 10.1371/journal.pone.0181926

**Published:** 2017-07-25

**Authors:** Santosh K. Patnaik, Eric D. Kannisto, Reema Mallick, Anil Vachani, Sai Yendamuri

**Affiliations:** 1 Department of Thoracic Surgery, Roswell Park Cancer Institute, Buffalo, New York, United States of America; 2 Department of Surgery, State University of New York, Buffalo, New York, United States of America; 3 Department of Surgery, University of Minnesota, Minneapolis, United States of America; 4 Department of Medicine, University of Pennsylvania, Philadelphia, Pennsylvania, United States of America; Institut de Pharmacologie Moleculaire et Cellulaire, FRANCE

## Abstract

At least seven studies have suggested that microRNA levels in whole blood can be diagnostic for lung cancer. We conducted a large bi-institutional study to validate this. Qiagen^®^ PAXgene^™^ Blood miRNA System was used to collect blood and extract RNA from it for 85 pathologic stage I-IV non-small cell lung cancer (NSCLC) cases and 76 clinically-relevant controls who had a benign pulmonary mass, or a high risk of developing lung cancer because of a history of cigarette smoking or age >60 years. Cases and controls were similar for age, gender, race, and blood hemoglobin and leukocyte but not platelet levels (0.23 and 0.26 million/μl, respectively; t test P = 0.01). Exiqon^®^ MiRCURY^™^ microarrays were used to quantify microRNAs in RNA isolates. Quantification was also performed using Taqman^™^ microRNA reverse transcription (RT)-PCR assays for five microRNAs whose lung cancer-diagnostic potential had been suggested in seven published studies. Of the 1,941 human mature microRNAs detectable with the microarray platform, 598 (31%) were identified as expressed and reliably quantified among the study's subjects. However, none of the microRNAs was differentially expressed between cases and controls (P >0.05 at false discovery rate <5% in test using empirical Bayes-moderated t statistics). In classification analyses with leave-one-out internal cross-validation, cases and controls could be identified by microRNA expression with 47% and 50% accuracy with support vector machines and top-scoring pair methods, respectively. Cases and controls did not differ for RT-PCR-based measurements of any of the five microRNAs whose biomarker potential had been suggested by seven previous studies. Additionally, no difference for microRNA expression was noticed in microarray-based microRNA profiles of whole blood of 12 stage IA-IIIB NSCLC cases before and three-four weeks after tumor resection. These findings show that whole blood microRNA expression profiles lack diagnostic value for high-risk screening of NSCLC, though such value may exist for selective sub-groups of NSCLC and control populations.

## Introduction

Primary cancer of the lung is the leading cause of cancer incidence and of cancer-associated mortality worldwide, with about 224,000 new cases and 158,000 deaths because of the disease projected to have occurred in year 2016 in United States of America alone [1]. Approximately 85% of primary lung cancer cases are of non-small cell variety, and 65%-70% of non-small cell lung cancers (NSCLC) are of adenocarcinoma (AC; 40%) and squamous cell carcinoma (SCC; 25%-30%) histological sub-type (e.g., [2]). Although 76%-93% of patients with lung cancer have a history of tobacco smoking (e.g., [3, 4]), with relative risks of 4–33 and 6–67 for AC or SCC observed respectively for 20–30 and 50–60 pack-years of cigarette smoking [5], not all smokers develop lung cancer. The risk of a cigarette smoker getting a diagnosis of lung cancer after 10 years of follow-up is estimated at about 5% [6]. Because clinical outcome of lung cancer is significantly improved by early diagnosis, screening of populations such as those of smokers and the elderly is important. Another population for which screening is important is that of individuals in whom a lung mass is identified in routine radiological tests. Such a mass has a 1%-70% chance of being cancer, depending on factors such as its size [7, 8].

A screening test for lung cancer that examines biomarkers in whole blood has the advantages of being non-invasive and not requiring separation of a specific component of blood such as serum or mononuclear cells. Findings of at least seven studies suggest that microRNAs may have a diagnostic biomarker utility in such a test. An altered whole blood microRNA expression profile in NSCLC was first noted in 2009 by Keller and colleagues, who examined 866 microRNAs in eight AC and seven SCC cases, and 19 healthy controls to identify 27 differentially expressed microRNAs between the cases and controls [9]. In a subsequent study, the same research group examined the expression of 863 microRNAs in blood of five each of AC and SCC cases, and 10 healthy subjects and identified 39 differentially expressed microRNAs [10]. In the two studies, cases and controls could be distinguished by their microRNA expression with 93%-100% sensitivity and 98%-100% specificity. Leidinger and colleagues compared whole blood expression of 863 microRNAs of 19 healthy individuals and of 24 individuals with chronic pulmonary disease against those of 28 cases of lung cancer (nine AC, 13 SCC), and noted differential expression of 70 and 250 microRNAs, respectively [11]. These researchers again observed differential expression of microRNAs– 24 of 1,205 that were examined–between groups of seven each of healthy individuals and NSCLC cases (five AC, one SCC) [12]. All sensitivity and specificity values were between 86% and 100% in both of their studies. We used Exiqon^®^ miRCURY^™^ microarrays to quantify levels of 1,282 microRNAs in whole blood of 22 AC cases and 23 clinically relevant controls, who either had a bengin pulmonary mass or were of age >50 years with a history of cigarette smoking of >20 pack-years [13]. The cases and controls differed for 96 microRNAs, whose expression could distinguish the two types of subjects with 91% sensitivity and 100% specificity. Unlike these five studies which utilized microarrays or RNA sequencing for an unbiased exploration of microRNA levels, two studies chose a few specific microRNAs to examine with reverse transcription (RT)-PCR assays. Only one microRNA, *let-7a-5p*, was evaluated by Jeong et al., and expression in blood of this microRNA was different between groups of 35 NSCLC cases (19 AC and 15 SCC) and 30 healthy individuals [14]. Ulivi et al. investigated 14 microRNAs and identified three to be differentially expressed between 86 NSCLC cases (63 AC and 22 SCC) and 24 healthy individuals [15]. MicroRNA expression could be used to categorize cases and controls in these two studies respectively with sensitivity of 90% and 70%, and specificity of 90% and 83%.

Discordance among these studies in identifying specific microRNAs as differentially expressed has been noted [13]. For instance, expression level of *let-7a-5p* was identified as signficantly different between cases and controls in two studies [9, 14] but not three others [10, 11, 13]. In five of the seven studies, total sample sizes were less than 60 and likely not adequate enough to reduce chance of false discovery of microRNA biomarkers below an acceptable limit. Also in five studies, the control group consisted of individuals in good health instead of those in a clinically relevant condition. Case and control cohorts were significantly different for age of their subjects in all but two studies [14, 15]. Thus, while these studies indicate a detectable effect of presence of NSCLC in the body on its blood microRNAs, the strength of this observation and its utilizable applicability to diagnosis of NSCLC remain questionable. We conducted the large bi-institutional study that is described here to robustly address these questions.

## Materials and methods

### Ethics statement

This study was approved by the Institutional Review Boards of Roswell Park Cancer institute (study identification number I 161709) and University of Pennsylvania (study identification number 806390). Study participants provided written informed consent.

### Estimation of power of study

Power and group size analysis was performed using the method of Ferreira and Zwinderman [16] with the SSPA [17] Bioconductor package (version 1.12.0) in R (version 2.14.1). Effect sizes used for this analysis were based on microRNA expression measurements obtained previously by us using fifth generation miRCURY^™^ microarrays (Exiqon^®^) for whole blood RNA of 23 cases of lung AC and 22 clinically relevant controls in a study whose finding suggested a diagnostic value of whole blood microRNAs for lung cancer [13]. The power analysis used the Student t null distribution, moderated t statistics and effect sizes calculated in comparison of cases and controls for differential microRNA expression with the limma Bioconductor package (version 3.10.3). The microRNA expression dataset that was examined had normalized expression values for the 395 microRNAs that were considered as expressed in that study. R code of the power and group size analysis is provided in S1 Text.

### Study population and blood collection

Study participants were 86 subjects with primary NSCLC (cases) and 75 subjects without any cancer (controls) who were evaluated at Hospital of University of Pennsylvania or Roswell Park Cancer Institute during 2010–2012. Peripheral venous blood (2.5 ml) was collected from the participants during hospital visits in a PAXgene^™^ Blood RNA tube (Qiagen^®^, Valencia, CA), which was then frozen at -20°C within two hours and then transferred to -80°C within a day for long-term storage. None of the cases received any treatment for cancer prior to blood collection, which was done within a month before lung cancer resection. For 12 cases, blood was also collected three to four weeks after the resection. Eighteen controls underwent surgery for a suspicious lung mass that on later pathological evaluation was found to be benign. Blood samples of these subjects were obtained within a month before surgery. The remaining 58 controls were chosen because of age >60 years or a history of cigarette smoking. Blood white blood cell (WBC) and platelet counts, and blood hemoglobin values at time-points closest to the time of blood collection for RNA isolation were collated from medical records. The time-points were before surgery for all but one case for whom it was immediately after surgery. For controls, blood counts and hemoglobin values could be obtained for 17 (74%); for six of them, the values were determined >90 days before blood collection for RNA isolation.

### Isolation of RNA from blood

PAXgene^™^ Blood miRNA kit (Qiagen^®^) and the protocol supplied by its manufacturer were used to extract total RNA from blood collected in PAXgene^™^ Blood RNA tubes. The tubes were thawed for 18–24 hours at 4°C before RNA extraction, which was done by one individual in nine batches during a six-week period. Cases and controls were equally represented in all batches. RNA extracted from 2.5 ml blood was collected in 80 μl of the BR5 buffer provided with the kit. Concentration and quality of RNA was assessed by absorbance spectrometry on NanoDrop^™^ 2000 (Thermo^®^, Waltham, MA) and by electrophoresis in a Bioanalyzer^™^ 2100 Eukaryote Total RNA Nano assay (Agilent^®^, Santa Clara, CA). RNA preparations were stored frozen at temperatures below -70°C and were used for microarray experiments within nine weeks.

### Microarray hybridization for microRNA quantification

Experiments were performed by Exiqon^®^ (Vedbaek, Denmark) as a commercial service using their seventh generation miRCURY^™^ microarray platform [18], which has 1,937 locked nucleic acid-containing DNA oligonucleotide probes that target 20 human non-microRNA small RNAs (20 probes), 25 human miRPlus^™^ (Exiqon^®^) mature microRNAs (25 probes), and 1,916 human mature microRNAs that are recorded in the miRBase database (1,892 probes). Thirty of the mature microRNA probes recognize multiple microRNAs (72 total; 2–6 per probe), some of which are also recognized by a second probe. Sample RNA (500 ng), spiked with 62 artificial small RNAs and then end-labeled with the Cy3-like Hy3^™^ dye using the miRCURY^™^ microRNA Power Labeling kit (Exiqon^®^), was hybridized to probes on a microarray along with 500 ng of reference RNA that had been similarly spiked with the artificial RNAs but labeled with the Cy5-like Hy5^™^ dye. The reference RNA was generated by combining the total RNA samples isolated from different human tissues that are provided with the FirstChoice^™^ Human Total RNA Survey Panel (product number AM6000, Ambion^™^, Austin, TX). After overnight hybridization, microarrays were washed, and then scanned and analyzed using ImaGene^®^ software (version 9; BioDiscovery^®^, Los Angeles, CA) to generate raw data files with Hy3^™^ and Hy5^™^ signal intensities. All labeled RNA samples were prepared in one batch. Hybridizations to microarrays of all labeled RNAs were performed in eight batches over four days, with RNA samples processed in the order in which they were prepared from blood.

### Microarray data processing

Raw microarray data was processed using well-established and commonly used methods that are also recommended by the microarray manufacturer. The same methods were also used by us in a study that utilized microarrays of the same manufacturer to suggest a diagnostic value of whole blood microRNAs for lung cancer [13]. Raw data files from all 181 microarray hybridizations that were performed were analyzed together. The 181 included three duplicate RNA samples and seven RNA samples that were later excluded from the study. Examination of signals, after within-array normalization and probe-level summarization as described later, for the 62 spiked-in RNAs indicated poor quality of data for one microarray (log_2_ ratio of medians of Hy3^™^ and Hy5^™^ signals below -0.8). Data from three other microarrays also had a poor quality as indicated by signals from empty spots on the microarrays (both Hy3^™^ and Hy5^™^ signal values above 32). Data from the remaining 177 microarrays was processed with the limma Bioconductor package. Background noise was subtracted from array signals using the convolution model-based normexp method with 10 as the offset value [19]. Signals were then normalized within a microarray using the global loess method with 1/3 as the span value [20], and then between microarrays using the quantile method [21]. Signals from multiple spots of a probe were summarized to a single value that was the mean when the maximum value was <1.5-fold of the minimum, and the median if otherwise. Signals were then filtered to exclude probes that did not have a manufacturer-provided annotation, or had a Hy3^™^ signal value <3-fold of the summarized signal value from empty spots of the same microarray in ≤25% of the 177 microarrays. The range, mean and standard deviation of the summarized signals for empty spots among the microarrays were 15.2–17.1, 15.4 and 0.23, respectively. The Hy3^™^ signal data-set was finally filtered to exclude data from probes that did not target human RNAs. Raw and processed microarray data is available in the Gene Expression Omnibus [22] repository with accession number GSE40738. R code used for microarray data processing is provided in S2 Text.

### Reverse transcription (RT)-PCR for small RNA quantification

TaqMan^™^ microRNA RT-PCR assays [23] that utilize stem-loop RT primers and real-time PCR were used. Assays were purchased from Applied Biosystems^®^ (Foster City, CA) for human *let-7a-5p*, *let-7g-5p*, *miR-93-3p*, *miR-126-3p*, *miR-630*, *miR-675*, *miR-942-5p*, *miR-1248*, and *miR-1284*. The identity numbers of the assays provided by the manufacturer were 0377, 2282, 2139, 2228, 1563, 2005, 2187, 2870 and 2903, respectively. A custom assay that has been described elsewhere [24] was used for the *RNU6-2* (*U6B*) small nucleolar house-heeping RNA. TaqMan^®^ microRNA reverse transcription kit (Applied Biosystems^®^) was used to reverse transcribe 200 ng RNA in a reaction of 15 ul as recommended by the manufacturer. Primers for all six analyte RNAs were included in an RT reaction; this multiplexing did not affect measurement of any analyte compared to RT that had only one, analyte-specific primer. Triplicate PCR reactions of 15 or 20 ul using RT reactions as template and FastStart^™^ Universal Probe Master (Rox) PCR master-mix (Roche^®^, Indianapolis, IN) were performed with real-time fluorometry on a 7900HT thermocycler (Applied Biosystems^®^). SDS software (version 2.4; Applied Biosystems^®^) was used with automatic baseline detection and a manually set cycle threshold of 0.05 to identify quantification cycle (C_q_) values, approximately inversely proportional to the log_2_ value of analyte RNA concentrations. The means of C_q_ values of the triplicate PCRs were used for further analysis. S2 Table lists these values. C_q_ values >36 were considered non-specific. RT-PCR assays of this study were performed by one individual during a ten-day period in batches of 16–17 RNA samples, with all PCR reactions for the batch performed on the same 384-well microplate. Every batch included water as negative control, which did not give any specific RT-PCR signal, and 200 ng of the reference human RNA of this study's microarray experiments for inter-batch calibration. Linear transformation was used for this calibration: for each analyte, all C_q_ values of a batch were adjusted by a value equal to the deviation of the intra-batch C_q_ value for the reference RNA from its inter-batch average. For normalization, microRNA C_q_ values were adjusted with intra-sample *RNU6-2* C_q_ values.

### Analysis of processed microarray data

Classification, correlation, differential expression, hierarchical clustering, and principal component analyses were performed on log_2_-transformed normalized Hy3^™^ microarray signal values for the 598 microRNAs identified as expressed in the study's cohorts. The prcomp function in R and TM4 [25] MeV software (version 4.8) were respectively used for the principal component and unsupervised hierarchical clustering analyses. Uncentered Pearson correlations, average linkages, and leaf order optimization were used for the clustering analyses. Differential expression analyses were performed using the limma package. In these analyses, a test like the t test but based on empirical Bayes-moderated t statistics was used. Classification analyses to examine the value of microRNA expression measurements in distinguishing cases and controls used classifiers that were identified by linear kernel support vector machines (SVM) and top-scoring pair (TSP) methods. In case of SVM, classifiers were tuned with a value set of 0.1, 0.2, 0.5, 1, 2, 5, 10, 20, 50 for *cost* [26], and consisted of 15 most differentially expressed microRNAs (genes) identified by the limma method. The CMA [27] (version 1.12.0) and tspair [28] (version 1.12.0) Bioconductor packages for R were used for the classification analyses. Cross-validations in these analyses used the leave-one-out (LOOCV) and Monte Carlo (MCCV) methods. MCCVs had 1,000 iterations and validation group sizes of 20 or 30.

#### Other

All statistical tests were two-tailed, and a P value below 0.05 was used to judge significance. To keep false discovery rate below 5% in multi-testing scenarios, P values were adjusted with the Benjamini-Hochberg method. Equal group variance was assumed in standard t tests. Correlation and receiver operating characteristic analyses, and Fisher and standard t tests were performed with Prism^™^ software (version 6.0 for Mac OS X; GraphPad^®^, La Jolla, CA).

## Results

### Statistical power of study

**Fig 1:**
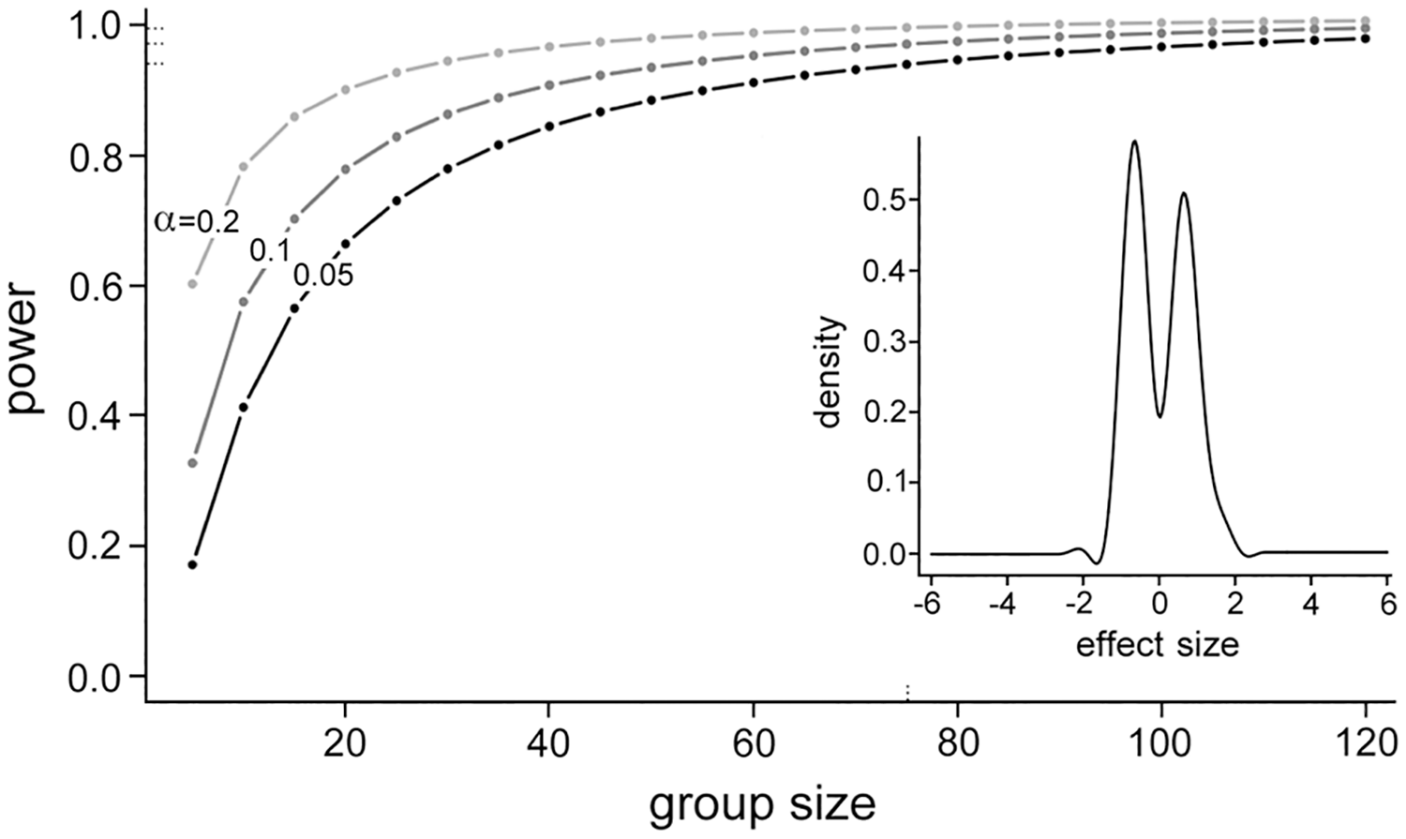
Power and group size analysis. Microarray-based microRNA quantifications of whole blood of 23 lung adenocarcinoma cases and 22 controls that had been obtained previously were analyzed. Effect sizes in case-control comparison were used to estimate study power at different group sizes and false positivity rates (α) of 0.05, 0.10 and 0.20. Inset shows density distribution of effect sizes in the microRNA data.

We based the estimation of this study's power on whole blood microRNA expression data that we had previously obtained for 23 lung AC cases of pathologic stage IA-IIIB and 22 clinically relevant controls [13]. The current study is nearly identical to the previous study for nature of the control cohort, collection and storage of blood, extraction of RNA from blood, and measurement of microRNAs in extracted RNA. As expected, density estimates of the effect sizes in the previous study's microRNA expression data had a bimodal distribution, indicating that levels of many microRNAs were higher among cases compared to controls while levels of many other microRNAs were lower (inset in Fig 1). The effect sizes were used to estimate and plot study power at different group sizes and false positivity rates (Fig 1). For case and control groups of 75 samples each, power values were calculated as 0.94 and 0.97 at false positivity rates of 5% and 10%, respectively, indicating that the current study is adequately powered to identify a difference in whole blood microRNA expression between cases and controls.

### Characteristics of case and control cohorts

This was a retrospective study in which subject selection was governed by availability of appropriate clinical information and blood specimens. All 85 cases and 76 controls who are examined in this study had health care or evaluation during years 2010–12 at Hospital of University of Pennsylvania (n = 87) or Roswell Park Cancer Institute (n = 74), two urban tertiary health-care centers. Clinical and demographic features of the case and control cohorts are provided in Table 1; detailed information for individual subjects are in S1 Table. The cases had a diagnosis of primary NSCLC, 45 (53%) and 33 (39%) of which respectively were of AC and SCC histology. The cancer was of pathologic stage I in 44% of the cases and III or IV in 30%. Most of the patients were of Caucasian ethnicity (84%) with a history of cigarette smoking (99%). The 76 controls were chosen for clinical relevance. Eighteen (24%) had surgical resection of a pulmonary mass that was later judged as benign after histopathological evaluation, with 13 (72%) of the resected masses diagnosed as granuloma. The other 58 controls had a high risk for developing lung cancer because of age >60 years (n = 40) and/or a history of cigarette smoking (n = 57). Among the 18 controls with benign lung mass, 11 were of age >60 years and 14 had a history of cigarette smoking. There was no significant difference between the case and control cohorts for age, gender, Caucasian ethnicity, or history of cigarette smoking (Table 1). Data on some blood parameters, prior to any surgical resection, could be obtained for 84 cases and 30 controls. The two cohorts did not differ significantly for blood hemoglobin level or white blood cell (WBC) count, but blood platelet count was significantly less by 12% among cases compared to controls (t test P = 0.01; Table 1).

**Table 1:** Characteristics of cohorts of the study.

	*Cases (n = 85)*	*Controls (n = 76)*	*P value*^a^
Mean age (years; range, SD^b^)	63.5 (41–83, 8.4)	61.1 (45–83, 8.7)	0.07
Male gender	42 (49%)	39 (51%)	0.88
Caucasian race	71 (84%)	67 (88%)	0.50
History of smoking	84 (99%)	71 (93%)	0.10
Stage of cancer (n = 85)			
I	37 (44%)		
II	22 (26%)		
III	14 (16%)		
IV	12 (14%)		
Histology of cancer (n = 85)			
Adenocarcinoma	45 (53%)		
Squamous cell carcinoma	33 (39%)		
Histology of benign lung nodule (n = 18)			
Granuloma	13 (72%)		
Hamartoma	2 (11%)		
Mean blood parameters (SD)	(n = 84)	(n = 30)	
White blood cells (x1000/μl)	6.7 (1.8)	7.7 (2.7)	0.15
Platelets (x1000/μl)	233.9 (81.1)	263.4 (68.8)	0.01
Hemoglobin (g/dl)	13.5 (1.4)	13.5 (1.3)	0.37
Mean RNA parameters (range, SD)			
Yield (μg)	8.3 (2.3–22.6; 3.6)	8.1 (3.0–24.8; 3.7)	0.74
RNA integrity number^c^	7.6 (5.5–9.0; 0.7)	7.5 (2.8–9.0; 0.9)	0.62
Absorbance 260 nm/280 nm	2.2 (2.1–2.5; 0.1)	2.2 (1.9–2.5; 0.1)	0.36
Absorbance 260 nm/230 nm	0.4 (0.1–1.0; 0.2)	0.4 (0.1–0.8; 0.2)	0.55

^a^In Fisher's exact test in case of categorical variables, and in two-tailed t tests assuming equal group variances in case of others.

^b^Standard deviation.

^c^Obtained by Bioanalyzer^™^ assay. Unknown for five samples.

### Microarray-based quantification of microRNAs in RNA isolated from whole blood

**Fig 2:**
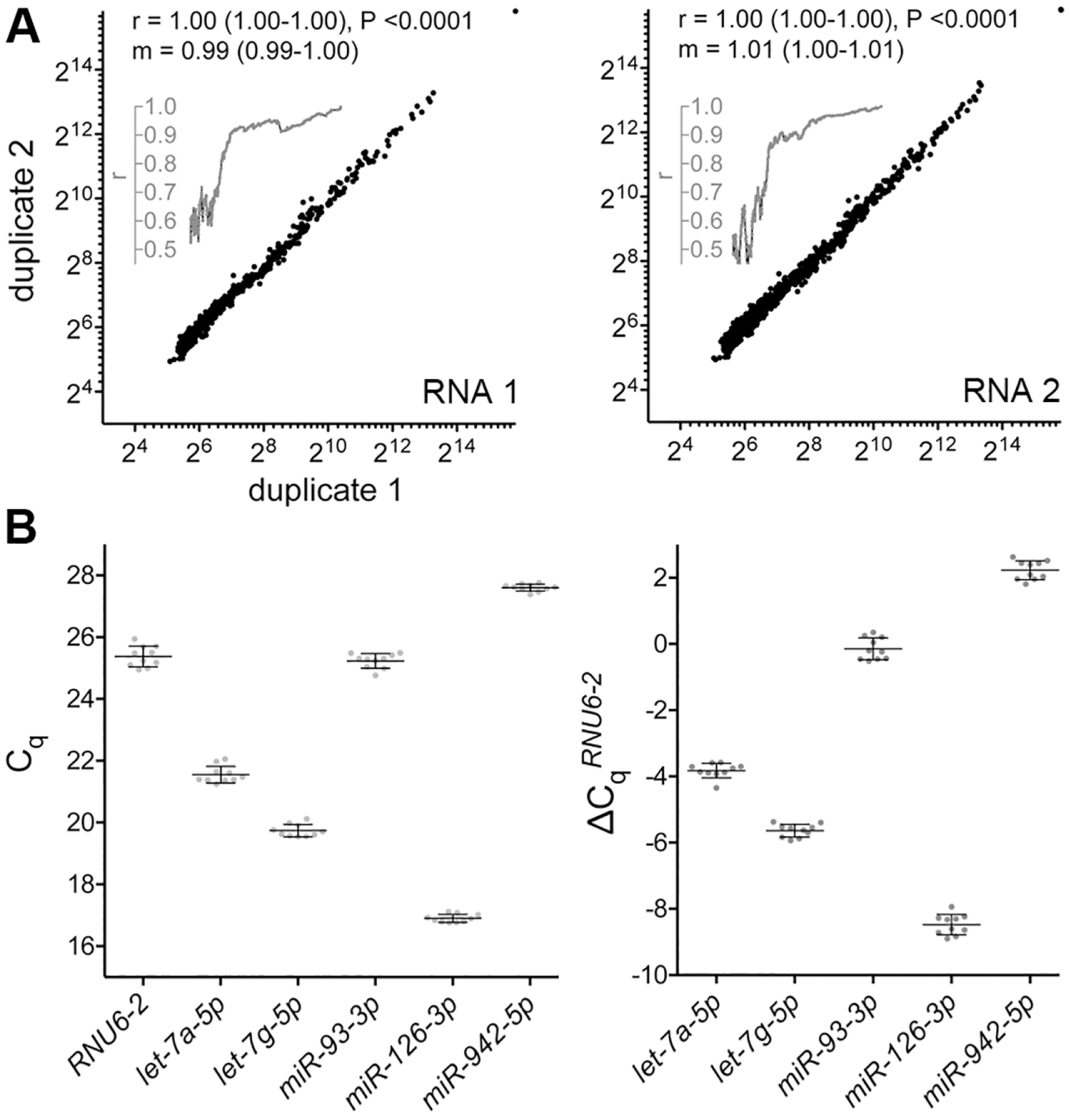
Technical replicability of microRNA quantification by microarray and reverse transcription (RT)-PCR. (**A**) The two scatter-plots show the inter-duplicate correlation of microRNA quantifications for two RNA samples (*RNA 1* and *2*) that were analyzed with microarrays in duplicate. Microarray signal values for the 598 expressed human microRNAs of this study are plotted for the two pairs of duplicates. Also shown are the coefficients of Pearson correlation (*r*, rounded to two decimal places), and their 95% confidence intervals and associated two-tailed P values, and the slopes (*m*) of the linear regression lines (ordinary least squares method) and their 95% confidence intervals. To depict the relatively poor correlation for microRNAs with low signals, a rolling window of width 99 along the X axis was used for calculating r at the mid-window abscissa and plots of these r values are shown in grey. (**B**). MicroRNAs *let-7a-5p*, *let-7g-5p*, *miR-93-3p*, *miR-126-3p*, and *miR-942-5p*, and the *RNU6-2* (*U6B*) small RNA were measured in a reference RNA in ten separate batches of RT-PCR assays. Raw (*left*) and *RNU6-2*-normalized (*right*) quantification cycle (C_q_) values from the ten assays, and their means and standard deviations are plotted.

To measure microRNA levels in whole blood of cases and controls, total RNA was first extracted from blood samples that had been collected in PAXgene^™^ Blood tubes, which contain reagents for cell lysis and RNA stabilization [29]. The blood samples had been obtained within a month before surgery for cases or controls who underwent operative procedures for removal of their lung masses. Amounts of RNA obtained from 2.5 ml blood of cases and controls were similar, with an overall mean of 8.2 μg (range = 2.3–24.8; standard deviation, SD = 3.6). RNAs of cases and controls were of similar quality, as suggested by their Bioanalyzer^™^ electropherograms and spectrophotometric absorbances at 230, 260 and 280 nm (Table 1 and S1 Table). There was a small but significant negative Pearson correlation between RNA yield and blood hemoglobin value (r = -0.2, P = 0.04) but not age, blood WBC count, or blood platelet count. As per the current miRBase microRNA sequence repository [30], humans have 2,588 known mature microRNAs. About 74% of them (1,916), and 25 proprietary Exiqon^®^ miRPlus^™^ mature microRNAs were quantified in the RNAs extracted from cases and controls with the seventh generation Exiqon^®^ miRCURY^™^ microarray platform, whose DNA oligonucleotide probes have locked nucleic acids for binding microRNAs with improved sensitivity and specificity [18]. All of the miRBase microRNAs that were detectable with the fifth generation of the miRCURY^™^ microarray platform that was used in our previous study [13] were also detectable with the seventh generation. Microarray signals for 598 microRNAs, detected by a total of 586 microarray probes, were reliably detected among at least a quarter of this study's samples. These 'expressed' microRNAs, which included 12 miRPlus^™^ microRNAs, were used for the analyses that are described here. Microarray assays were done in duplicate for three RNA samples, and inter-duplicate correlation of measurements of the 598 expressed microRNAs was good for all three samples (Pearson r >0.99; Fig 2A). About 13% of the miRBase microRNAs that were identified as expressed in our previous study [13] were not among the 598 expressed microRNAs of this study. To assess the effect of blood parameters on microRNA levels, Spearman correlation analyses were performed. Modest but significant correlations, with coefficient values between 0.35 and 0.50, were noted for levels of four, five and two microRNAs respectively with blood hemoglobin, platelet count and WBC count values. These microRNAs included *miR-23a-3p* and *miR-223-3p*, which are known to be highly expressed in platelets and myeloid cells, respectively [31, 32].

### Validation of microarray-based microRNA quantifications using RT-PCR

**Fig 3:**
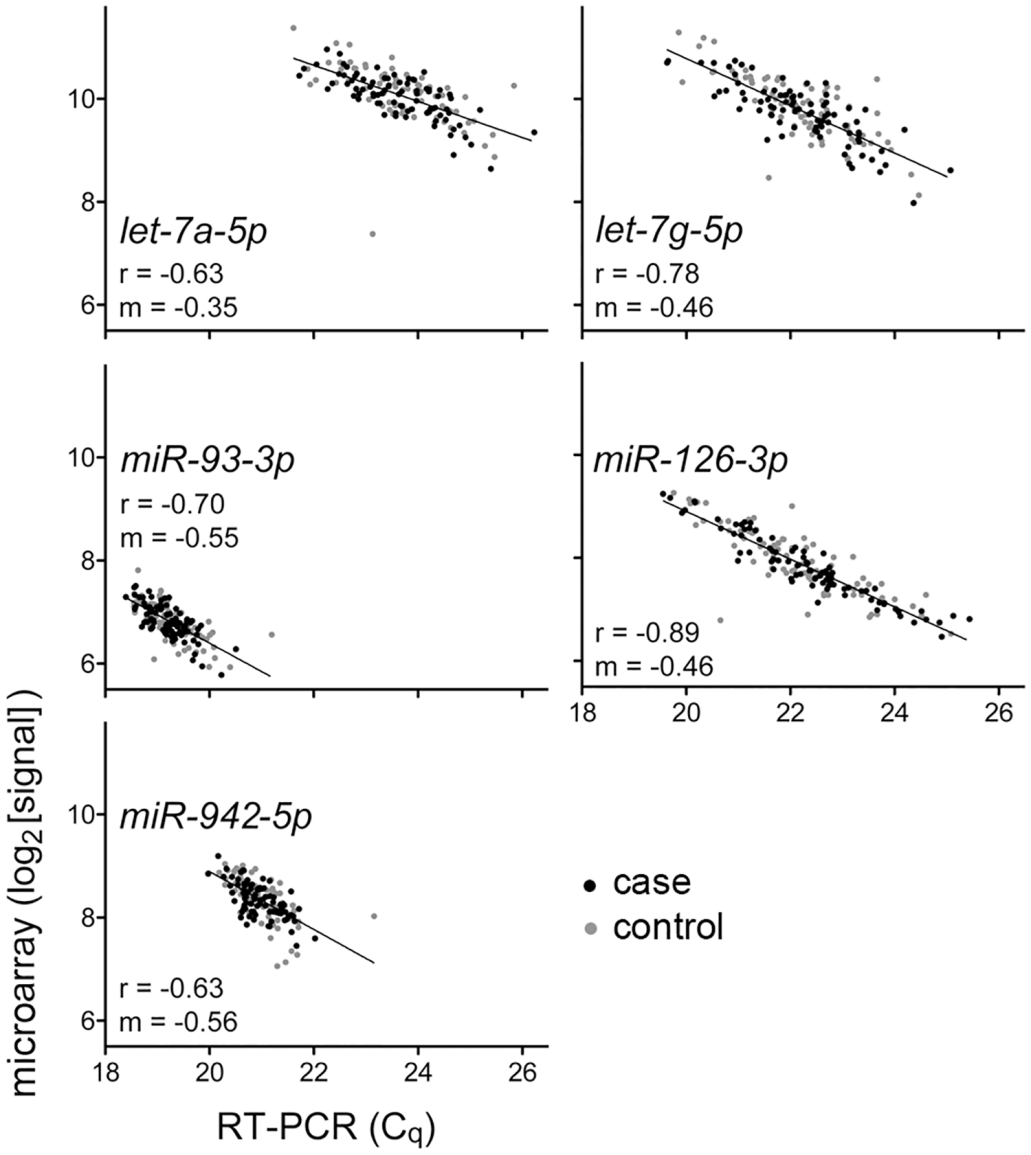
Confirmation of microarray-based microRNA quantifications by reverse transcription (RT)-PCR. Scatter-plots show measurements of five microRNAs in 85 cases (*black*) and 76 controls (*gray*) by RT-PCR (*C*_*q*_, quantification cycle value) or microarray (log_2_-transformed signal value). Also shown are the best-fitting lines (least squares method) and their slopes (*m*), and Pearson correlation coefficients (*r*).

To assess the accuracy of the microRNA expression data-set that was generated using microarrays, levels of five microRNAs in all 161 RNA samples of cases and control were also measured with TaqMan^®^ microRNA RT-PCR assays [23]. The microRNAs that were examined were *let-7a-5p*, *let-7g-5p*, *miR-93-3p*, *miR-126-3p*, and *miR-942-5p*. These microRNAs were selected so that the RT-PCR data can also be used to evaluate their potential as lung cancer biomarkers that has been suggested by other studies. A reference RNA was used to calibrate measurements obtained in the ten batches of RT-PCR assays that were performed. The assays had good replicability across batches (Fig 2B), and all five microRNAs were detected in all 161 samples (S2 Table). As has been observed previously (e.g., [13, 33]), the RT-PCR-based measurements had a wider range (1.2–2.1x) compared to those obtained by microarray. There was good Pearson correlation between the sets of measurements obtained by RT-PCR and microarray for all five microRNAs (-0.90 < r < -0.63), indicating validity of the microarray-based microRNA quantification (Fig 3). Such correlation coefficient values between TaqMan^™^ RT-PCR and miRCURY^™^ microarray platforms are noted in numerous studies (e.g., [33]).

### Similarity of whole blood microRNA profiles and cases and controls

**Fig 4:**
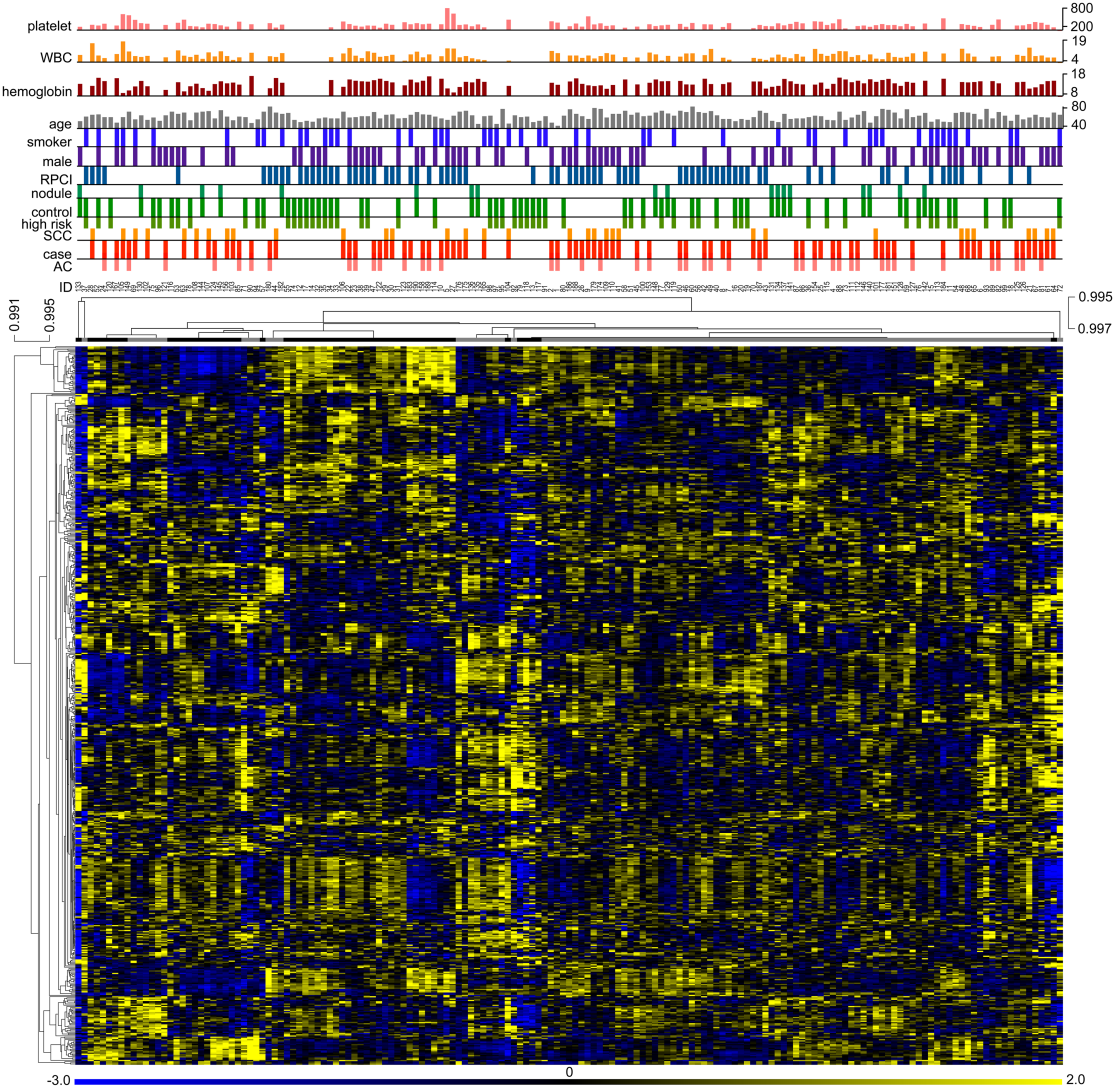
Whole blood microRNA expression among cases and controls. A heat map, with its pseudo-color scale underneath, depicts Z-scaled log_2_-transformed microarray signal values for the study's 598 expressed microRNAs among its 161 cases and controls. Unsupervised hierarchical clustering is used to order the samples and microRNAs, dendrograms for which are drawn based on uncentered Pearson correlations, average linkages, and leaf order optimization. Node heights are indicated by the scales next to the dendrograms. Lower-level detail of the sample dendrogram beyond a distance threshold is not shown, with elements on the nodes below the threshold instead depicted as one cluster (*black* and *grey bars*). Graphs at the top of the figure indicate cohort membership (case or control) of the samples, and their age (in years), gender, institution (*RPCI*, Roswell Park Cancer Institute), and blood hemoglobin (g/dl), platelet count (x10^3^/ul), and white blood cell (*WBC*) count (x10^3^/ul) values (if known). Adenocarcinoma (*AC*) or squamous cell carcionoma (*SCC*) histology of cancer, presence of a benign nodule in controls, or their being at high risk to develop lung cancer, and sample identities (*ID*) are also indicated.

To compare cases and controls for microRNA levels, a differential expression analysis was performed. Empirical Bayes-moderated t statistics calculated by the limma Bioconductor package [34] were used in the analysis, and P values obtained for case-control comparisons were adjusted by the Benjamini-Hochberg method to keep false discovery rate in the multi-testing below 5%. This differential expression analysis revealed that levels of none of the 598 microRNAs were significantly different between the case and control cohorts (all adjusted P >0.05). S3 Table lists the 19 microRNAs for which the adjusted P value was between 0.05 and 0.15. Statistically significant differential expression of microRNAs was not seen if subjects with cancer of only AC histology (n = 45) were included in the case cohort. Three microRNAs had differential expression when cases were of only SCC histology (n = 33). Similarity of whole blood microRNA profiles and cases and controls that was suggested by the differential expression analysis was also noticeable in an unsupervised hierarchical clustering analysis of the case and control samples by their microRNA levels. The analysis used Pearson correlations among values for the set of 598 expressed microRNAs and did not reveal a good separation of cases and controls. This is depicted in Fig 4, which also has a heat map of relative microRNA expression among the study subjects. The lack of segregation of cases and controls by their microRNA expression was also noticeable in visualization of the top three principal components of the microRNA data (S1A Fig).

### Inability of whole blood microRNA expression profiles to distinguish cases from controls

To test if the whole blood microRNA profiles contained information from a subset of microRNAs that could be used to distinguish cases from controls, classification analyses with internal cross-validation were performed. In these analyses, microRNA data of a sub-group of subjects was used to develop a classifier and the ability of the classifier to accurately identify the remaining subjects as case or control by their microRNA expression was evaluated. Size of sub-group used for classifier development was 160 in LOOCV cross-validation. In MCCV cross-validation, the size was 131; MCCV was iterated a thousand times with randomized subject selection. Two separate methods were employed to develop classifiers: SVM with linear kernel, and TSP. These methods were chosen for their simplicity and because they were used in our previous study on diagnostic value of whole blood microRNAs for lung cancer [13]. SVM classifiers had 15 variables, microRNAs that were identified as most differentially expressed between cases and controls as per the empirical Bayes-moderated t test implemented in the limma Bioconductor package. TSP classifiers, which are independent of differential expression of microRNAs, had two variables (microRNAs) [35]. In LOOCV with the SVM classification method, accuracy, sensitivity and specificity for categorization of cases and controls by their microRNA expression were quantified to be 47%, 47% and 57%, respectively. With the TSP method, the values were 50%, 49% and 51%, respectively. The poor performance characteristics of both SVM and TSP classifiers were also noted in MCCV (Table 2). To examine if classification was better for a specific histology of NSCLC, the analyses were performed for subjects that included only AC or SCC cases. Sizes of test groups in MCCV were reduced from 30 to 20 for these analyses. As shown in Table 2, restricting cases to one cancer sub-type, either AC or SCC, significantly improved sensitivity of SVM classifiers from 47% to 83%-88% in both LOOCV and MCCV. However, accuracy and specificity measurements remained poor. Results of these classification analyses were thus concordant with those of differential expression, unsupervised clustering, and principal component analyses, which indicated the absence of a strong association between existence of lung cancer and whole blood microRNA expression.

**Table 2:** Performance in classification analyses of microRNA expression profiles^a^.

*Subjects*	*N*^b^	*Classifier type*^c^	*Validation method*^d^	*Accuracy (%)*	*Sensitivity (%)*	*Specificity (%)*
All cases; all controls	85; 76	SVM	LOOCV	47	47	57
			MCCV	56 (8)	53 (15)	61 (13)
		TSP	LOOCV	50	49	51
			MCCV	54 (8)	55 (18)	53 (16)
Adenocarcinoma cases; all controls	45; 76	SVM	LOOCV	62	84	24
			MCCV	61 (10)	83 (13)	27 (18)
		TSP	LOOCV	65	51	87
			MCCV	50 (9)	53 (20)	47 (29)
Squamous cell carcinoma cases; all controls	33; 76	SVM	LOOCV	64	87	12
			MCCV	68 (9)	88 (10)	23 (19)
		TSP	LOOCV	36	51	0
			MCCV	60 (9)	66 (17)	50 (27)

^a^Mean, with standard deviation in parentheses, of values obtained in 1,000 iterations are shown for MCCV.

^b^Sizes of the two groups whose subjects are examined in the analysis.

^c^SVM–linear kernel support vector machines; TSP–top-scoring pair.

^d^LOOCV–leave-one-out cross validation; MCCV–Monte Carlo cross-validation.

### Cancer resection does not affect whole blood microRNA expression

**Fig 5:**
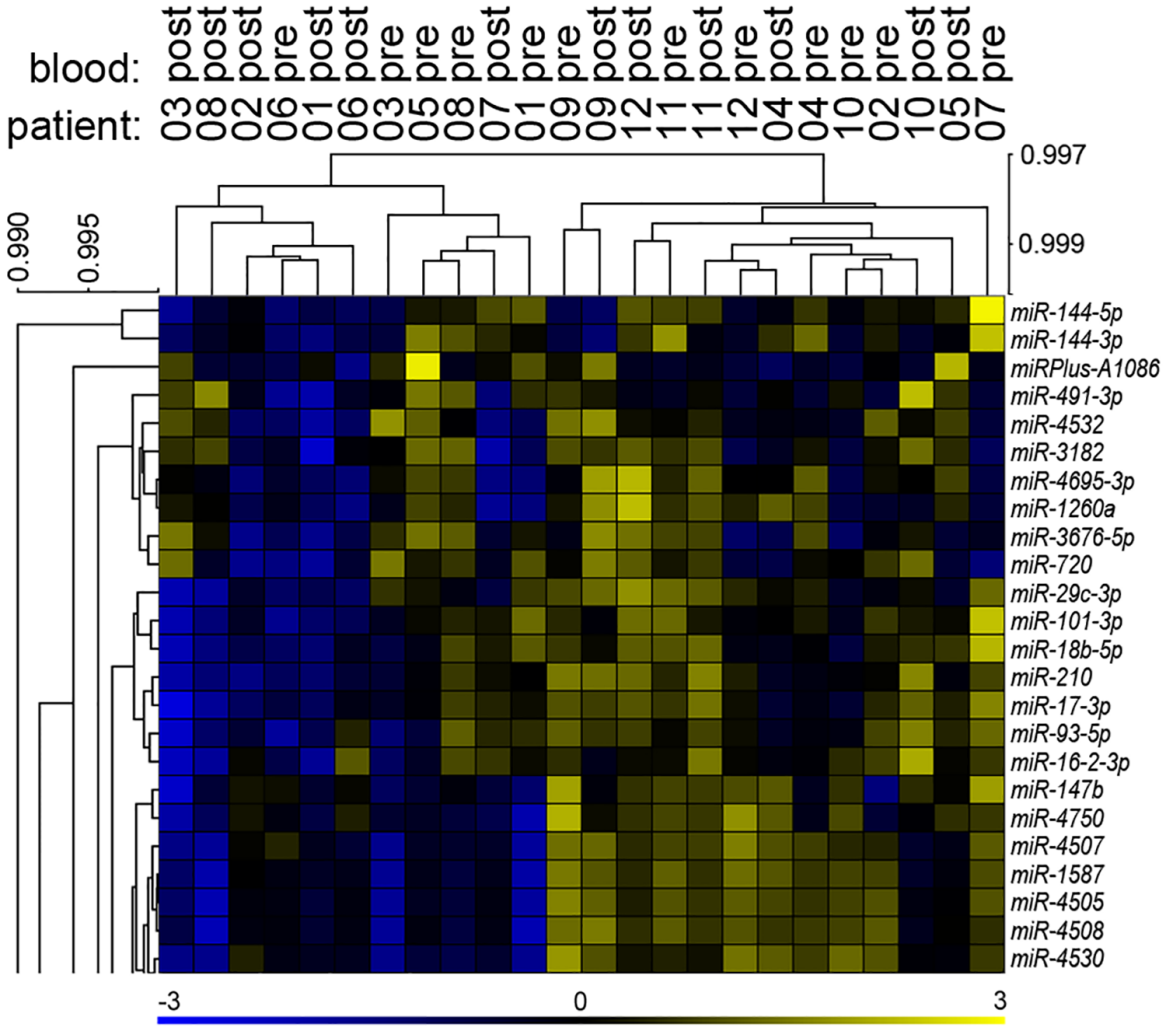
Unsupervised hierarchical clustering of whole blood microRNA profiles of pre- and post-resection samples. Twelve non-small cell lung cancer cases of pathologic stage I before (*pre*) and three to four weeks after (*post*) tumor resection were analyzed. Microarray-based measurements the study's 598 expressed microRNAs are used for the clustering. Uncentered Pearson correlations, with average linkages used for joining clusters, are used to draw the leaf order-optimized microRNA and sample dendrograms. Node heights are indicated by the scales next to the dendrograms. Patient and time-point identifiers are shown above the sample dendrogram. The heat map is truncated (like the microRNA dendrogram), and shows inter-sample Z-scaled expression values of 24 microRNAs. A pseudo-color scale for the values is provided below the heat map.

It is conceivable that an effect of presence of lung cancer on blood microRNA expression may disappear after surgical removal of the cancer, and this may be noticed in comparison of microRNA levels before and after surgery. For 12 cases of this study, whole blood samples that had been collected three to four weeks after surgical resection of lung cancer were available, and microRNA expression profiles of these samples were obtained along with the other samples of this study. Histology of lung cancer was AC for eight of these cases and SCC in the others (S1 Table). Eight of the tumors were of pathologic stage I. A paired differential expression analysis of the pre- and post-surgery microRNA profiles of the 12 cases, which used the empirical Bayes-moderated t test implemented in the limma Bioconductor package, showed that surgery had no significant effect on the blood level of any of the 598 expressed microRNAs of this study. Furthermore, segregation of the microRNA profiles by time of blood collection was not seen in either unsupervised hierarchical clustering or principal component analyses (Fig 5 and S1B Fig).

### Invalidation of previously suggested microRNA biomarkers by RT-PCR

Results of seven studies that have suggested a value for whole blood microRNA expression for lung cancer were examined to choose ten microRNAs that were noted by the studies for differential expression or for being a constituent of microRNA classifiers. The concordance among the findings of these studies has been noted to be poor [13]. Diagnostic values were observed for *let-7a-5p*, *let-7g-5p*, *miR-126-3p*, *miR-1248*, *miR-675*, *miR-942-5p*, and *miR-93-3p* respectively in only two [9, 14], two [9, 10], one [9], two [10, 13], two [11, 13], two [12, 13], and four of the seven studies [9, 10, 12, 13]. A diagnostic value for *miR-630* and *-1284* was suggested by us [13] but either not examined or not noticed in the other six studies. As mentioned earlier, microarray-based measurements of blood level of none of the nine microRNAs was different between cases and controls of this study. To strengthen this invalidation of the previously suggested microRNA biomarkers, measurements of the microRNAs obtained with a different method were analyzed. RT-PCR-based measurements could be obtained for five microRNAs; the other four could not be detected in whole blood RNA with the assays that were used (C_q_ >36). The microRNA measurements for each sample were normalized against the sample's measurement value for *RNU6-2* (*U6B*). *RNU6-2* is probably the most commonly used normalizer for RT-PCR-based microRNA measurements. It was used as the normalizer for RT-PCR data in the study of Jeong et al. [14], one of the two RT-PCR-based studies that have suggested a diagnostic value of whole blood microRNAs for lung cancer. For the five detectable microRNAs (*let-7a-5p*, *let-7g-5p*, *miR-93-3p*, *miR-126-3p*, and *miR-942)*, areas under curve were <0.56 in receiver operating characteristic analyses. Levels of none of these microRNAs were different between cases and controls (t test P >0.25; Fig 3). The lack of a significant difference was also seen when only those with AC or only those with SCC were included in the case cohort.

## Discussion

This study investigated the plausible usefulness of microRNA expression levels in whole blood as biomarkers for screening NSCLC in clinically relevant populations. Levels of about 75% of the 2,588 known human mature microRNAs were determined in blood of 86 NSCLC cases and 75 controls from two different institutions (Table 1). The controls, individuals with history of cigarette smoking or diagnosed radiologically with a lung mass, represented populations for which there is a need for a screening test for lung cancer. Cases and controls were matched for age, ethnicity, gender, and history of cigarette smoking, and for blood hemoglobin values and WBC counts. The study had good statistical power (Fig 1), and it utilized molecular assays with good technical replicability to determine microRNA levels that were partially validated (Figs 2 and 3). Examination of the whole blood microRNA levels in a variety of analyses–classification, differential expression, principal components, unsupervised clustering–failed to reveal any difference between cases and controls (Fig 4 and S1A Fig). A difference in levels of cases and controls for five microRNAs whose utility as lung cancer-diagnostic whole blood biomarkers has been suggested in seven published studies was also not seen when the microRNA levels in whole blood were quantified by a different method (RT-PCR). Additionally, any effect of resection of cancer on whole blood microRNA expression was not noticed (Fig 5 and S1B Fig). These findings suggest that whole blood microRNAs do not have a diagnostic value for screening NSCLC in a population with history of chronic smoking or of old age. It remains possible that whole blood microRNAs do possess a diagnostic value for specific sub-types of NSCLC and/or other types of screening population, and that they have a biomarker value for other aspects of NSCLC besides diagnosis, such as indication of responsiveness to immunotherapy.

Only about 75% of known human mature microRNAs were examined by microarray in this study, and only 598 of the examined microRNAs were detected and quantified among its samples. It is thus possible that one or more microRNAs with true diagnostic value were missed by the study. Besides this issue of sensitivity, the likely lack of absolute specificity of at least some of the probes of the microarray platform [18] could also have contributed to the failure to detect the diagnostic value of whole blood microRNAs. Aside from differences in the nature of study populations, differences in sensitivity and specificity among different platforms to quantify microRNAs (e.g., [36, 37]) likely explains to some degree the discordance among the seven previous studies that have suggested a diagnostic value of whole blood microRNAs for lung cancer [13]. In five of the seven studies, total sample sizes were small (<60), and a false discovery of microRNA biomarkers in these studies is a possibility. Case and control cohorts differed significantly for age in all but two of the seven studies [14, 15]. This difference for age, and that all cases were of AC sub-type and were recruited at one institution may explain the discrepancy between the findings of our current and previous studies.

Different methods of processing and normalization of microRNA expression data too can have significant effect on results of microRNA expression data analyses (e.g., [38, 39]). In this study, we utilized *RNU6-2* RNA measurements for normalizing for microRNA measurements obtained by RT-PCR. *RNU6-2* is a commonly used normalizer and was used so in the study of Jeong et al. [14], one of the two RT-PCR-based studies that have suggested a diagnostic value of whole blood microRNAs for lung cancer. The other study, by Ulivi and colleagues used other small RNAs for normalization [15]. The microarray data that were obtained in our study was processed using well-established and commonly used methods that are also recommended by the microarray manufacturer. The same methods were also used by us in a study that utilized microarrays of the same manufacturer to suggest a diagnostic value of whole blood microRNAs for lung cancer [13]. We did not bias our study by attempting to analyze the microarray- or RT-PCR-based microRNA measurements with different normalization methods to identify a method with which a biomarker value of whole blood microRNAs could be identified. Instead, we chose data processing and normalization methods that are used commonly and have been used in previous studies on whole blood microRNAs in lung cancer. The suitability of the methods that we used is also indicated by the observation of reasonably good correlation of the microarray- and RT-PCR-based normalized microRNA measurement values (Fig 3).

Our study evaluated microRNA expression in whole blood samples, and its findings have no relevance to biomarker values for NSCLC of microRNA levels in specific components of blood such as plasma and mononuclear cells. MicroRNAs in whole blood exist both extracellularly, within microvesicles that arise from all parts of the body, or bound to circulating proteins, and intracellularly within blood cells such as leukocytes and any circulating cancer cell population. Changes in whole blood microRNA expression profiles have been associated with both non-cancerous diseases, such as myocardial infarction [40] and sarcoidosis [41], and cancer of tissues besides lung such as breast [42] and ovary [43]. While these changes may arise from diseased tissue, they may also have a complex causation such as a disease-predisposing genetic constitution that affects microRNA expression, or a specific type of immune response by the body that changes circulating immune cell sub-populations. Blood microRNA expression profiles also reflect the physiological state of the body, as suggested by studies that have shown their correlations with age [44], blood pressure [45], diurnal rhythm [46], gender [47], mental anxiety [48], physical stress [49], etc. Thus, while the presence of NSCLC can per se have an effect on microRNAs in blood, detection of such an effect on the microRNA analytes whose levels are influenced by many other factors may be difficult or impossible.

## Supporting information

S1 Fig Plots of principal components of whole blood microRNA expression data. Scatter-plots of the top three principal components (*PC 1–3*) are shown. The axis labels include values of proportions contributed by the components to data variance. Log_2_-transformed microarray signal values for the study's 598 expressed microRNAs were analyzed. (**A**). Principal component plots for the 85 cases and 76 controls of the study, sub-groups of which are indicated by shape and color of symbols. (**B**). Principal component plots for microRNA expression before (*pre*) and three-four weeks after (*post*) surgical resection of cancer for 12 non-small cell lung cancer cases. A unique color is used to display each case.
(TIF)

S1 Table Clinical, demographic and RNA characteristics of individuals of the study. (DOC)

S2 Table RNA measurements obtained by reverse transcription (RT)-PCR. (DOC)

S3 Table Expression of microRNAs in the case and control cohorts with adjusted P <0.15 in one differential expression analysis. (DOC)

S1 Text R code used for estimating power of study. (DOC)

S2 Text R code used for processing of raw microarray data. (DOC)

S3 Text R code used for one set of classification analyses. (DOC)
