# Supplementary figures and images for: Whole blood microRNA expression may not be useful for screening non-small cell lung cancer
Source: PLoS One. 2017 Jul 25;12(7):e0181926. doi: 10.1371/journal.pone.0181926 (PMC5526508; doi:10.1371/journal.pone.0181926)

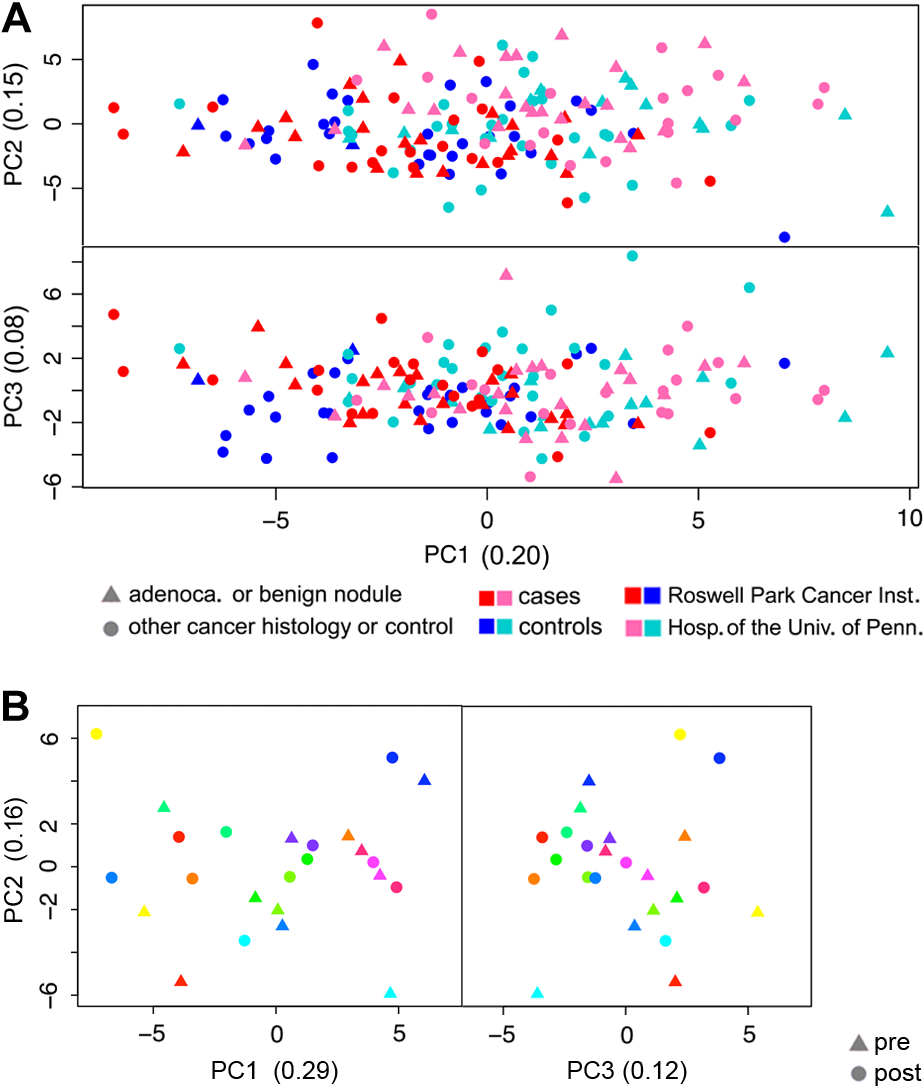

Supplement: S1 Fig — Scatter-plots of the top three principal components (PC 1–3) are shown. The axis labels include values of proportions contributed by the components to data variance. Log2-transformed microarray signal values for the study's 598 expressed microRNAs were analyzed. (A). Principal component plots for the 85 cases and 76 controls of the study, sub-groups of which are indicated by shape and color of symbols. (B). Principal component plots for microRNA expression before (pre) and three-four weeks after (post) surgical resection of cancer for 12 non-small cell lung cancer cases. A unique color is used to display each case. (TIF) [file pone.0181926.s001.tif]
