# Supplementary material for: Whole blood microRNA expression may not be useful for screening non-small cell lung cancer
Source: PLoS One. 2017 Jul 25;12(7):e0181926. doi: 10.1371/journal.pone.0181926 (PMC5526508; doi:10.1371/journal.pone.0181926)
Supplement: S1 Table — (DOC) [file pone.0181926.s002.doc]

**S1 Table.** *Clinical, demographic and RNA characteristics of individuals of the study.^a,b^*

| *RNA identity* | *Individual identity* | *Cohort* | *Center* | *Age (years)* | *Gender* | *Race* | *Smoking status* | *Histology* | *Cancer stage* | *Blood white blood celll count (x10^3^/ul)* | *Blood hemoglobin level (g/dl)* | *Blood platelet count (x10^3^/ul)* | *RNA yield (ug)* | *RNA absorbance 260 nm:280 nm* | *RNA absorbance 260 nm:230 nm* | *RNA integrity number* |
| --- | --- | --- | --- | --- | --- | --- | --- | --- | --- | --- | --- | --- | --- | --- | --- | --- |
| 1 | 1 | L | R | 41 | F | W | P | A | IA | 9.6 | 12.6 | 327 | 5.0 | 2.19 | 0.33 | 7.1 |
| 2 | 2 | L | R | 48 | M | W | P | A | IV | 11.2 | 13.6 | 368 | 6.8 | 2.13 | 0.43 | 7.4 |
| 4 | 3 | L | R | 53 | F | W | P | A | IV | 9.1 | 13.1 | 304 | 5.6 | 2.19 | 0.39 | 7.3 |
| 5 | 4 | L | R | 54 | M | W | C | S | IIIB | 13.1 | 10.8 | 820 | 5.8 | 2.16 | 0.14 | 7.4 |
| 6 | 5 | L | R | 55 | M | W | P | U | IIIA | 3.4 | 14.4 | 187 | 5.1 | 2.19 | 0.25 | 8.0 |
| 7 | 6 | L | R | 56 | F | W | P | A | IIA | 4.8 | 13.8 | 312 | 15.1 | 2.13 | 0.82 | 8.1 |
| 8 | 7 | L | R | 56 | F | N | C | N | IV | 6.2 | 13.0 | 187 | 10.6 | 2.13 | 0.54 | 7.6 |
| 9 | 8 | L | R | 56 | F | W | C | S | IIIA | 7.9 | 12.4 | 530 | 7.9 | 2.15 | 0.51 | 7.5 |
| 10 | 9 | L | R | 57 | F | W | C | A | IB | 7.3 | 15.7 | 274 | 3.7 | 2.20 | 0.28 | 7.8 |
| 11 | 104 | H | R | 49 | F | W | C |  |  |  |  |  | 7.5 | 2.23 | 0.29 | 7.8 |
| 12 | 105 | H | R | 49 | F | W | C |  |  |  |  |  | 7.6 | 2.15 | 0.47 | 8.1 |
| 13 | 106 | H | R | 49 | F | W | P |  |  |  |  |  | 10.8 | 2.18 | 0.64 | 6.2 |
| 14 | 107 | H | R | 50 | M | W | P |  |  |  |  |  | 8.0 | 2.13 | 0.46 | 7.1 |
| 15 | 108 | H | R | 50 | F | W | C |  |  |  |  |  | 4.8 | 2.39 | 0.26 | 6.0 |
| 16 | 109 | H | R | 51 | M | W | P |  |  |  |  |  | 16.5 | 2.12 | 0.74 |  |
| 17 | 110 | H | R | 52 | M | W | C |  |  |  |  |  | 6.2 | 2.16 | 0.36 | 7.0 |
| 18 | 111 | H | R | 52 | F | W | C |  |  |  |  |  | 6.4 | 2.15 | 0.40 | 6.5 |
| 19 | 112 | H | R | 54 | F | W | P |  |  |  |  |  | 8.7 | 2.14 | 0.27 | 6.2 |
| 20 | 113 | H | R | 56 | F | W | P |  |  |  |  |  | 10.8 | 2.19 | 0.34 | 6.7 |
| 21 | 10 | L | R | 59 | F | W | P | S | IIA | 13.6 | 10.7 | 280 | 4.6 | 2.29 | 0.32 | 7.1 |
| 22 | 11 | L | R | 59 | M | W | C | A | IV | 13.4 | 15.3 | 280 | 10.1 | 2.11 | 0.63 | 7.6 |
| 23 | 12 | L | R | 60 | F | W | P | A | IA | 7.8 | 14.7 | 228 | 8.6 | 2.10 | 0.52 | 6.8 |
| 24 | 13 | L | R | 60 | M | B | P | A | IIA | 5.9 | 14.5 | 295 | 3.2 | 2.31 | 0.11 | 7.2 |
| 25 | 14 | L | R | 62 | F | W | P | L | IIB | 9.1 | 13.7 | 356 | 6.7 | 2.24 | 0.28 | 7.4 |
| 26 | 15 | L | R | 62 | F | W | P | A | IA | 6.1 | 13.1 | 197 | 8.0 | 2.15 | 0.46 | 6.4 |
| 27 | 16 | L | R | 63 | F | W | P | N | IV | 12.9 | 8.8 | 622 | 4.4 | 2.23 | 0.28 | 6.5 |
| 28 | 17 | L | R | 65 | M | W | P | S | IA | 16.8 | 12.1 | 256 | 2.3 | 2.52 | 0.10 | 6.4 |
| 29 | 18 | L | R | 65 | F | W | P | S | IV | 7.4 | 14.6 | 294 | 9.3 | 2.18 | 0.20 | 6.6 |
| 30 | 19 | L | R | 66 | M | W | P | S | IB | 8.4 | 13.8 | 259 | 7.7 | 2.13 | 0.43 | 5.5 |
| 31 | 114 | H | R | 57 | F | W | C |  |  |  |  |  | 8.0 | 2.14 | 0.50 | 6.8 |
| 32 | 115 | H | R | 57 | M | W | P |  |  |  |  |  | 5.5 | 2.20 | 0.36 | 7.0 |
| 33 | 116 | H | R | 57 | M | W | C |  |  |  |  |  | 6.1 | 2.16 | 0.24 | 6.9 |
| 34 | 117 | H | R | 57 | M | W | C |  |  | 6.7 | 15.0 | 170 | 6.2 | 2.24 | 0.32 | 7.5 |
| 35 | 118 | H | R | 59 | M | W | C |  |  |  |  |  | 7.6 | 2.21 | 0.32 | 6.9 |
| 36 | 119 | H | R | 60 | F | W | C |  |  |  |  |  | 7.8 | 2.19 | 0.40 | 7.0 |
| 37 | 120 | H | R | 61 | M | W | C |  |  |  |  |  | 5.0 | 2.13 | 0.34 |  |
| 38 | 121 | H | R | 62 | M | W | P |  |  | 6.0 | 14.4 | 197 | 13.2 | 2.13 | 0.45 | 8.2 |
| 39 | 122 | H | R | 62 | F | N | P |  |  | 10.9 | 14.2 | 235 | 6.3 | 2.13 | 0.33 | 6.9 |
| 40 | 123 | H | R | 64 | F | W | P |  |  |  |  |  | 10.4 | 2.13 | 0.43 | 8.0 |
| 41 | 20 | L | R | 67 | M | W | P | S | IIIA |  |  |  | 3.6 | 2.31 | 0.26 |  |
| 42 | 21 | L | R | 69 | F | W | P | A | IB | 8.1 | 15.7 | 272 | 4.6 | 2.26 | 0.33 | 6.9 |
| 43 | 22 | L | R | 69 | F | W | P | S | IIIA | 4.6 | 10.7 | 213 | 11.5 | 2.14 | 0.51 | 8.4 |
| 44 | 23 | L | R | 70 | M | W | P | S | IIA | 6.8 | 16.0 | 169 | 5.8 | 2.19 | 0.23 | 6.5 |
| 45 | 24 | L | R | 70 | M | W | P | A | IA | 6.4 | 12.8 | 196 | 3.6 | 2.25 | 0.24 | 6.3 |
| 46 | 25 | L | R | 71 | F | W | P | A | IV | 9.3 | 12.8 | 193 | 6.4 | 2.14 | 0.33 | 6.6 |
| 47 | 26 | L | R | 71 | M | W | P | A | IV | 9.9 | 14.9 | 224 | 10.2 | 2.11 | 0.61 | 6.8 |
| 48 | 27 | L | R | 71 | F | W | P | S | IV | 12.8 | 14.0 | 278 | 5.1 | 2.23 | 0.18 | 6.8 |
| 49 | 28 | L | R | 73 | F | W | P | A | IA | 12.7 | 13.0 | 271 | 11.3 | 2.08 | 0.64 | 7.8 |
| 50 | 29 | L | R | 74 | M | W | P | A | IV | 7.0 | 14.6 | 288 | 6.6 | 2.17 | 0.16 | 8.1 |
| 51 | 124 | H | R | 65 | M | W | P |  |  |  |  |  | 9.4 | 2.16 | 0.45 | 7.8 |
| 52 | 125 | H | R | 66 | F | W | C |  |  | 7.9 | 16.1 | 233 | 4.7 | 2.16 | 0.14 | 7.9 |
| 53 | 126 | H | R | 67 | F | W | P |  |  | 11.6 | 13.9 | 320 | 5.2 | 2.24 | 0.15 | 8.2 |
| 54 | 127 | H | R | 69 | F | W | C |  |  |  |  |  | 6.0 | 2.30 | 0.16 | 7.4 |
| 55 | 128 | H | R | 69 | M | W | P |  |  |  |  |  | 19.4 | 2.11 | 0.62 |  |
| 56 | 129 | H | R | 71 | F | W | P |  |  |  |  |  | 7.3 | 2.19 | 0.47 | 8.2 |
| 57 | 130 | H | R | 72 | F | W | C |  |  |  |  |  | 3.0 | 2.52 | 0.16 | 7.9 |
| 58 | 131 | H | R | 74 | M | W | P |  |  | 6.7 | 15.3 | 161 | 4.3 | 2.36 | 0.23 | 6.8 |
| 59 | 132 | H | R | 77 | M | W | N |  |  | 6.9 | 14.1 | 286 | 6.5 | 2.24 | 0.25 | 8.0 |
| 60 | 133 | H | R | 83 | F | W | P |  |  | 9.5 | 12.7 | 318 | 4.9 | 2.24 | 0.26 | 6.7 |
| 61 | 30 | L | P | 59 | M | W | P | S | IIIA | 6.4 | 14.5 | 257 | 7.7 | 2.24 | 0.48 | 7.4 |
| 63 | 31 | L | P | 70 | F | B | P | S | IIB | 7.7 | 9.3 | 426 | 10.1 | 2.14 | 0.45 | 7.6 |
| 64 | 32 | L | P | 70 | M | W | P | S | IA | 6.4 | 14.7 | 175 | 11.2 | 2.20 | 0.35 | 7.8 |
| 65 | 33 | L | P | 67 | M | A | P | S | IIIA | 7.1 | 14.0 | 223 | 8.8 | 2.19 | 0.31 | 7.8 |
| 67 | 34 | L | P | 65 | M | W | P | S | IB | 7.5 | 11.4 | 349 | 8.0 | 2.16 | 0.33 | 8.7 |
| 68 | 35 | L | P | 70 | F | W | C | S | IIB | 9.4 | 13.8 | 253 | 5.7 | 2.21 | 0.36 | 7.4 |
| 69 | 36 | L | P | 55 | M | W | C | S | IIIA | 7.5 | 11.8 | 421 | 8.9 | 2.22 | 0.18 | 8.5 |
| 70 | 37 | L | P | 60 | M | W | P | S | IIA | 6.2 | 14.3 | 151 | 10.2 | 2.18 | 0.34 | 8.3 |
| 71 | 134 | H | P | 60 | M | W | P |  |  |  |  |  | 3.8 | 2.22 | 0.27 | 7.7 |
| 72 | 135 | H | P | 65 | M | W | C |  |  |  |  |  | 6.8 | 2.11 | 0.38 | 5.9 |
| 73 | 136 | H | P | 54 | F | W | C |  |  | 10.9 | 15.3 | 134 | 7.6 | 2.20 | 0.26 | 6.9 |
| 74 | 137 | H | P | 54 | M | B | P |  |  |  |  |  | 9.9 | 2.18 | 0.38 | 7.4 |
| 75 | 138 | H | P | 59 | M | W | C |  |  |  |  |  | 6.0 | 2.24 | 0.16 | 8.9 |
| 76 | 139 | H | P | 63 | M | W | C |  |  |  |  |  | 14.6 | 2.17 | 0.52 | 8.4 |
| 77 | 140 | H | P | 74 | F | W | P |  |  | 4.3 | 14.4 | 236 | 9.5 | 2.17 | 0.33 | 8.4 |
| 78 | 141 | H | P | 53 | F | W | P |  |  | 6.8 | 11.9 | 217 | 14.0 | 2.15 | 0.66 | 8.3 |
| 79 | 142 | H | P | 60 | M | W | C |  |  |  |  |  | 11.5 | 2.19 | 0.62 | 8.4 |
| 80 | 143 | H | P | 63 | M | W | P |  |  |  |  |  | 13.4 | 2.14 | 0.51 | 8.6 |
| 81 | 38 | L | P | 60 | F | W | N | A | IA | 7.9 | 12.9 | 325 | 4.1 | 2.27 | 0.28 | 7.8 |
| 82 | 39 | L | P | 71 | F | W | P | A | IB | 7.3 | 13.4 | 431 | 9.8 | 2.12 | 0.61 | 8.8 |
| 85 | 40 | L | P | 61 | M | W | P | A | IV | 6.6 | 12.7 | 360 | 5.0 | 2.22 | 0.30 | 8.1 |
| 86 | 41 | L | P | 57 | F | W | P | A | IA | 7.0 | 12.8 | 304 | 4.9 | 2.21 | 0.31 | 7.4 |
| 87 | 42 | L | P | 57 | F | B | P | A | IIB | 8.9 | 13.1 | 352 | 9.1 | 2.13 | 0.48 | 9.0 |
| 88 | 43 | L | P | 72 | M | W | P | A | IB | 11.0 | 16.2 | 437 | 9.4 | 2.13 | 0.39 | 8.1 |
| 89 | 44 | L | P | 57 | F | W | P | A | IA | 7.0 | 14.5 | 255 | 10.3 | 2.17 | 0.43 | 8.5 |
| 90 | 45 | L | P | 47 | M | W | P | A | IA | 9.8 | 17.0 | 272 | 5.0 | 2.07 | 0.34 | 8.4 |
| 91 | 144 | H | P | 62 | M | W | C |  |  |  |  |  | 6.4 | 2.14 | 0.35 | 8.4 |
| 92 | 145 | H | P | 64 | M | W | P |  |  |  |  |  | 4.9 | 2.20 | 0.17 | 8.3 |
| 93 | 146 | H | P | 78 | M | W | P |  |  |  |  |  | 24.8 | 2.09 | 0.81 | 9.0 |
| 94 | 147 | H | P | 55 | F | W | C |  |  |  |  |  | 5.5 | 2.18 | 0.26 | 8.5 |
| 95 | 148 | H | P | 78 | F | W | P |  |  |  |  |  | 12.4 | 2.11 | 0.63 | 8.7 |
| 96 | 149 | H | P | 52 | M | W | P |  |  |  |  |  | 5.7 | 2.24 | 0.37 | 8.2 |
| 97 | 150 | H | P | 45 | F | W | C |  |  |  |  |  | 9.9 | 2.09 | 0.52 | 8.5 |
| 98 | 151 | H | P | 63 | M | B | C |  |  |  |  |  | 10.9 | 1.89 | 0.66 | 8.6 |
| 99 | 152 | H | P | 63 | M | W | P |  |  |  |  |  | 8.3 | 2.23 | 0.47 | 7.6 |
| 100 | 153 | H | P | 62 | M | W | C |  |  |  |  |  | 8.0 | 2.14 | 0.50 | 8.4 |
| 101 | 46 | L | P | 62 | M | W | C | S | IIB | 5.6 | 14.1 | 245 | 8.6 | 2.24 | 0.51 | 7.2 |
| 102 | 47 | L | P | 54 | F | B | P | S | IIIB | 7.6 | 11.9 | 293 | 5.6 | 2.30 | 0.20 | 8.2 |
| 103 | 48 | L | P | 53 | M | W | P | S | IIA | 4.7 | 13.7 | 89 | 8.5 | 2.18 | 0.53 | 7.4 |
| 104 | 49 | L | P | 46 | F | W | C | S | IV | 4.1 | 12.1 | 422 | 3.9 | 2.30 | 0.28 | 6.8 |
| 105 | 50 | L | P | 60 | M | B | C | S | IIIA | 18.1 | 8.5 | 603 | 10.2 | 2.21 | 0.56 | 8.2 |
| 106 | 51 | L | P | 72 | M | W | P | S | I | 9.3 | 11.6 | 366 | 6.2 | 2.20 | 0.21 | 7.0 |
| 107 | 52 | L | P | 73 | M | W | P | S | IA | 10.1 | 9.6 | 290 | 7.1 | 2.24 | 0.43 | 7.5 |
| 108 | 53 | L | P | 70 | F | W | P | S | IIA | 8.4 | 13.2 | 201 | 7.1 | 2.20 | 0.27 | 7.0 |
| 109 | 54 | L | P | 59 | M | W | P | S | IIA | 9.6 | 10.6 | 220 | 15.9 | 2.13 | 0.34 | 8.1 |
| 110 | 55 | L | P | 67 | F | W | P | S | IIB | 8.5 | 12.5 | 238 | 17.6 | 2.13 | 0.85 | 7.8 |
| 111 | 56 | L | P | 59 | M | B | P | A | IIA | 6.8 | 13.9 | 99 | 9.9 | 2.21 | 0.23 | 7.9 |
| 112 | 57 | L | P | 65 | M | W | P | A | IIIA | 8.1 | 14.7 | 216 | 12.5 | 2.16 | 0.60 | 7.9 |
| 113 | 154 | H | P | 55 | M | W | C |  |  |  |  |  | 6.6 | 2.24 | 0.23 | 7.1 |
| 114 | 155 | H | P | 61 | M | W | C |  |  |  |  |  | 4.6 | 2.32 | 0.32 | 6.4 |
| 115 | 156 | H | P | 54 | F | W | P |  |  | 7.6 | 10.6 | 248 | 5.9 | 2.20 | 0.40 | 7.2 |
| 116 | 157 | H | P | 72 | M | W | P |  |  |  |  |  | 5.1 | 2.23 | 0.30 |  |
| 117 | 158 | H | P | 52 | F | W | C |  |  |  |  |  | 7.5 | 2.20 | 0.30 | 7.5 |
| 118 | 159 | H | P | 61 | F | B | P |  |  |  |  |  | 8.8 | 2.11 | 0.49 | 6.8 |
| 119 | 160 | H | P | 63 | F | W | C |  |  |  |  |  | 7.2 | 2.18 | 0.39 | 7.2 |
| 120 | 161 | H | P | 60 | F | W | P |  |  |  |  |  | 5.8 | 2.30 | 0.19 | 7.7 |
| 121 | 58 | L | P | 64 | M | W | P | A | IA | 6.6 | 10.1 | 248 | 12.4 | 2.13 | 0.64 | 7.8 |
| 122 | 59 | L | P | 57 | M | W | P | A | IIA | 6.9 | 15.7 | 159 | 5.8 | 2.18 | 0.40 | 7.7 |
| 123 | 60 | L | P | 58 | M | B | P | A | IA | 4.2 | 12.4 | 338 | 16.3 | 2.08 | 0.89 | 8.2 |
| 124 | 61 | L | P | 57 | F | W | P | A | IIA | 7.2 | 13.0 | 281 | 5.8 | 2.19 | 0.34 | 5.9 |
| 125 | 62 | L | P | 65 | F | W | C | A | IIA | 8.4 | 13.9 | 231 | 6.4 | 2.15 | 0.34 | 8.2 |
| 126 | 63 | L | P | 55 | F | B | P | A | IIIA | 7.6 | 14.1 | 238 | 6.4 | 2.19 | 0.31 | 7.8 |
| 127 | 64 | L | P | 60 | M | W | P | A | IA | 9.5 | 13.6 | 210 | 13.3 | 2.14 | 0.38 | 7.8 |
| 128 | 86 | N | P | 57 | F | W | P | G |  | 6.3 | 12.1 | 206 | 7.3 | 2.24 | 0.38 | 8.0 |
| 129 | 87 | N | P | 68 | F | B | N | G |  | 7.6 | 14.5 | 259 | 5.8 | 2.23 | 0.35 | 7.7 |
| 130 | 88 | N | P | 64 | F | W | N | G |  | 6.8 | 14.6 | 308 | 6.2 | 2.22 | 0.25 | 7.8 |
| 131 | 89 | N | P | 52 | M | W | P | H |  | 6.4 | 11.6 | 208 | 4.4 | 2.19 | 0.12 | 7.3 |
| 133 | 90 | N | P | 51 | F | W | P | G |  | 8.3 | 13.1 | 189 | 14.4 | 2.18 | 0.32 | 2.8 |
| 134 | 91 | N | P | 49 | F | W | C | G |  | 8.5 | 12.1 | 303 | 5.2 | 2.28 | 0.32 | 7.8 |
| 135 | 92 | N | P | 45 | M | W | P | G |  | 5.1 | 15.6 | 269 | 11.0 | 2.18 | 0.49 | 7.9 |
| 136 | 93 | N | P | 64 | F | W | N | G |  | 5.8 | 13.8 | 210 | 6.7 | 2.16 | 0.44 | 8.2 |
| 137 | 94 | N | P | 67 | M | B | P | G |  | 6.7 | 15.6 | 237 | 9.4 | 2.14 | 0.52 | 8.1 |
| 140 | 95 | N | P | 48 | M | B | C | G |  | 8.7 | 15.4 | 189 | 7.6 | 2.21 | 0.49 | 7.4 |
| 141 | 96 | N | P | 56 | M | B | P | H |  | 5.9 | 14.4 | 289 | 5.0 | 2.23 | 0.25 | 7.7 |
| 142 | 97 | N | P | 69 | M | W | P | M |  | 10.6 | 14.7 | 297 | 9.1 | 2.16 | 0.47 | 7.2 |
| 144 | 98 | N | P | 72 | F | W | N | G |  | 5.5 | 11.0 | 175 | 9.2 | 2.12 | 0.56 | 7.4 |
| 145 | 99 | N | P | 75 | F | B | P | P |  | 10.9 | 14.4 | 239 | 9.0 | 2.15 | 0.56 | 7.1 |
| 146 | 100 | N | P | 73 | M | W | P | G |  | 4.7 | 13.4 | 234 | 4.5 | 2.36 | 0.20 | 8.1 |
| 148 | 101 | N | P | 70 | M | W | P | G |  | 8.6 | 15.3 | 213 | 5.1 | 2.19 | 0.35 | 7.1 |
| 149 | 65 | L | P | 72 | F | B | P | A | IIB | 10.8 | 9.8 | 567 | 5.8 | 2.15 | 0.39 | 7.8 |
| 151 | 66 | L | P | 70 | F | B | P | A | IA | 6.8 | 13.4 | 252 | 5.8 | 2.20 | 0.25 | 8.1 |
| 153 | 67 | L | P | 63 | M | W | C | A | IIB | 10.0 | 13.6 | 276 | 6.0 | 2.18 | 0.37 | 7.5 |
| 154 | 68 | L | P | 63 | F | B | C | A | IIA | 12.7 | 12.7 | 370 | 8.1 | 2.17 | 0.55 | 8.1 |
| 155 | 69 | P | P | 61 | F | W | C |  |  |  |  |  | 6.7 | 2.15 | 0.44 | 7.5 |
| 156 | 69 | L | P | 61 | F | W | C | S | IA | 7.4 | 13.4 | 196 | 8.3 | 2.15 | 0.45 | 7.3 |
| 157 | 72 | P | P | 48 | M | W | C |  |  |  |  |  | 6.5 | 2.14 | 0.26 | 7.6 |
| 158 | 70 | L | P | 66 | M | W | P | A | IA | 6.5 | 14.7 | 276 | 8.0 | 2.11 | 0.56 | 7.5 |
| 159 | 67 | P | P | 63 | M | W | C |  |  |  |  |  | 3.7 | 2.21 | 0.30 | 7.4 |
| 161 | 71 | P | P | 62 | M | B | C |  |  |  |  |  | 5.2 | 2.15 | 0.42 | 5.3 |
| 162 | 66 | P | P | 70 | F | B | P |  |  |  |  |  | 7.5 | 2.15 | 0.52 | 7.2 |
| 163 | 68 | P | P | 63 | F | B | C |  |  |  |  |  | 6.2 | 2.16 | 0.45 | 7.4 |
| 164 | 65 | P | P | 72 | F | B | P |  |  |  |  |  | 4.8 | 2.23 | 0.23 | 7.7 |
| 165 | 71 | L | P | 62 | M | B | C | S | IA | 4.6 | 14.1 | 171 | 9.1 | 2.18 | 0.20 | 7.3 |
| 166 | 70 | P | P | 66 | M | W | P |  |  |  |  |  | 6.4 | 2.18 | 0.45 | 8.0 |
| 167 | 72 | L | P | 48 | M | W | C | A | IIIA | 8.9 | 15.9 | 235 | 5.5 | 2.15 | 0.32 | 7.5 |
| 169 | 74 | P | R | 74 | F | W | P |  |  |  |  |  | 13.2 | 2.12 | 0.67 | 7.2 |
| 170 | 76 | P | R | 78 | F | W | C |  |  |  |  |  | 6.9 | 2.18 | 0.44 | 7.8 |
| 172 | 77 | P | R | 80 | F | W | P |  |  |  |  |  | 8.4 | 2.12 | 0.56 | 7.7 |
| 173 | 83 | P | R | 74 | M | W | P |  |  |  |  |  | 23.8 | 2.10 | 1.06 | 7.7 |
| 174 | 73 | L | R | 78 | F | W | P | A | IA | 7.5 | 12.4 | 302 | 13.8 | 2.15 | 0.46 | 7.7 |
| 175 | 74 | L | R | 74 | F | W | P | S | IA | 7.4 | 14.1 | 266 | 9.3 | 2.13 | 0.32 | 7.3 |
| 176 | 75 | L | R | 72 | F | W | P | R | IIA | 6.5 | 11.5 | 248 | 22.6 | 2.14 | 1.01 | 7.4 |
| 177 | 76 | L | R | 78 | F | W | C | A | IB | 5.3 | 15.0 | 281 | 7.0 | 2.23 | 0.44 | 8.1 |
| 179 | 77 | L | R | 80 | F | W | P | S | IA | 5.7 | 12.0 | 270 | 15.8 | 2.11 | 0.84 | 8.2 |
| 180 | 78 | L | R | 83 | M | W | P | A | IA | 7.3 | 10.9 | 308 | 3.8 | 2.21 | 0.23 | 6.7 |
| 181 | 79 | L | R | 76 | F | W | P | A | IA | 9.9 | 15.6 | 246 | 12.4 | 2.17 | 0.73 | 8.2 |
| 182 | 102 | N | R | 67 | M | W | C | G |  | 9.7 | 13.6 | 260 | 10.8 | 2.14 | 0.66 | 8.3 |
| 183 | 80 | L | R | 65 | M | W | C | L | IB | 8.6 | 15.2 | 276 | 10.7 | 2.09 | 0.64 | 8.1 |
| 184 | 81 | L | R | 50 | F | W | C | A | IB | 9.3 | 15.7 | 465 | 7.5 | 2.14 | 0.28 | 8.3 |
| 186 | 82 | L | R | 77 | M | W | P | S | IB | 7.8 | 14.3 | 206 | 10.3 | 2.13 | 0.29 | 8.3 |
| 187 | 83 | L | R | 74 | M | W | P | A | IB | 5.4 | 14.3 | 253 | 11.3 | 2.13 | 0.59 | 8.8 |
| 188 | 84 | L | R | 67 | F | W | C | L | IIIA | 7.8 | 15.8 | 296 | 12.6 | 2.12 | 0.50 | 8.4 |
| 189 | 85 | L | R | 65 | M | W | P | A | IB | 8.7 | 16.9 | 291 | 7.0 | 2.11 | 0.20 | 8.3 |
| 190 | 103 | N | R | 71 | F | W | P | F |  | 10.7 | 13.8 | 308 | 7.8 | 2.12 | 0.51 | 8.3 |

^a^Characteristics of individual cases (n = 86) and controls (n = 75) of the study, and of the total 173 blood RNA preparations are noted. For 12 cases, RNAs were extracted from blood samples that were collected both before and after lung cancer resection. Missing values indicate unavailability or non-applicability.

^b^Notations used for different variables:

- Center: P – Hospital of the University of Pennsylvania; R – Roswell Park Cancer Institute
- Cohort: L – lung cancer; H – control at high risk for lung cancer; N – control with bening lung nodule; P – after resection of lung cancer
- Gender: F – female; M – male
- Histology of tumor/nodule: A – adenocarcinoma; F – sub-pleural fibrosis; G – granuloma; H – hamartoma; L – large cell carcinoma; M – amyloidosis; N – neuroendocrine cancer; P – pneumonia; R – sarcomatoid carcinoma; S – squamous cell carcinoma; U – poorly differentiated non-small cell lung cancer
- Race: A – Asian; B – African American; N – Native American; W – White
- Smoking status: C – current cigarette smoker; N – never smoked cigarettes; P – smoked cigarettes in the past
