# Supplementary material for: Whole blood microRNA expression may not be useful for screening non-small cell lung cancer
Source: PLoS One. 2017 Jul 25;12(7):e0181926. doi: 10.1371/journal.pone.0181926 (PMC5526508; doi:10.1371/journal.pone.0181926)
Supplement: S2 Table — (DOC) [file pone.0181926.s003.doc]

**S2 Table.** *RNA measurements obtained by reverse transcription (RT)-PCR.^a^*

| *RNA^b^* | *Batch^c^* | *RNU6-2* | *let-7a-5p* | *let-7g-5p* | *miR-93-3p* | *miR-126-3p* | *miR-942-5p* |
| --- | --- | --- | --- | --- | --- | --- | --- |
| Reference | 1 | 25.705 | 21.360 | 19.770 | 25.247 | 16.809 | 27.661 |
| Reference | 2 | 25.174 | 21.247 | 19.553 | 25.422 | 16.899 | 27.621 |
| Reference | 3 | 25.096 | 21.394 | 19.548 | 25.304 | 16.770 | 27.617 |
| Reference | 4 | 25.236 | 21.482 | 19.709 | 25.284 | 16.930 | 27.674 |
| Reference | 5 | 24.992 | 21.405 | 19.602 | 24.761 | 16.761 | 27.388 |
| Reference | 6 | 25.689 | 21.976 | 19.985 | 25.491 | 17.078 | 27.726 |
| Reference | 7 | 25.945 | 22.053 | 20.113 | 25.485 | 17.109 | 27.756 |
| Reference | 8 | 25.504 | 21.644 | 19.627 | 24.991 | 16.790 | 27.466 |
| Reference | 9 | 24.951 | 21.374 | 19.581 | 25.304 | 17.012 | 27.576 |
| Reference | 10 | 25.475 | 21.602 | 19.905 | 25.043 | 16.840 | 27.571 |
| 1 | 1 | 27.538 | 22.460 | 20.792 | 18.666 | 20.903 | 20.858 |
| 2 | 1 | 27.078 | 22.169 | 20.565 | 18.858 | 20.945 | 21.246 |
| 4 | 1 | 27.903 | 24.138 | 22.607 | 19.238 | 22.669 | 21.624 |
| 5 | 1 | 27.459 | 23.028 | 20.962 | 19.692 | 19.603 | 21.726 |
| 6 | 1 | 30.789 | 23.974 | 22.347 | 18.795 | 22.066 | 20.722 |
| 7 | 1 | 28.758 | 23.173 | 22.114 | 19.473 | 22.656 | 21.287 |
| 8 | 1 | 27.364 | 23.165 | 21.581 | 19.208 | 22.435 | 21.284 |
| 9 | 1 | 27.735 | 22.750 | 21.146 | 19.222 | 20.865 | 21.545 |
| 10 | 1 | 27.023 | 22.313 | 20.308 | 18.890 | 19.832 | 20.660 |
| 11 | 1 | 27.964 | 23.835 | 22.240 | 18.928 | 21.582 | 21.761 |
| 12 | 1 | 29.572 | 22.242 | 20.364 | 18.672 | 20.125 | 20.555 |
| 13 | 1 | 29.553 | 23.762 | 21.890 | 19.107 | 20.830 | 21.365 |
| 14 | 1 | 28.223 | 23.119 | 21.549 | 19.045 | 20.560 | 20.748 |
| 15 | 1 | 26.500 | 23.333 | 22.066 | 18.644 | 22.633 | 20.657 |
| 16 | 1 | 28.883 | 23.452 | 21.850 | 19.120 | 21.948 | 21.335 |
| 17 | 1 | 27.433 | 22.096 | 20.532 | 18.933 | 20.953 | 20.986 |
| 18 | 2 | 28.136 | 23.254 | 21.883 | 18.847 | 22.271 | 20.315 |
| 19 | 2 | 28.572 | 23.402 | 21.830 | 19.054 | 21.265 | 20.802 |
| 20 | 2 | 29.270 | 23.914 | 22.423 | 19.324 | 21.770 | 21.224 |
| 21 | 2 | 29.244 | 24.307 | 22.932 | 19.295 | 21.654 | 21.585 |
| 22 | 2 | 29.086 | 23.898 | 22.001 | 19.458 | 21.327 | 21.435 |
| 23 | 2 | 28.262 | 23.347 | 21.867 | 19.114 | 21.118 | 21.181 |
| 24 | 2 | 27.829 | 22.336 | 21.071 | 18.743 | 21.231 | 20.186 |
| 25 | 2 | 28.276 | 23.494 | 22.201 | 19.368 | 22.311 | 20.569 |
| 26 | 2 | 27.946 | 23.137 | 21.730 | 19.531 | 20.915 | 20.994 |
| 27 | 2 | 27.098 | 22.275 | 20.632 | 19.369 | 20.170 | 21.464 |
| 28 | 2 | 27.830 | 22.526 | 20.872 | 18.766 | 21.065 | 20.290 |
| 29 | 2 | 28.291 | 23.600 | 21.663 | 19.908 | 20.606 | 22.043 |
| 30 | 2 | 28.503 | 23.679 | 21.726 | 19.355 | 21.058 | 21.630 |
| 31 | 2 | 28.509 | 23.034 | 21.563 | 19.445 | 20.807 | 21.416 |
| 32 | 2 | 27.237 | 22.221 | 20.694 | 19.236 | 19.760 | 20.868 |
| 33 | 2 | 27.712 | 23.141 | 21.535 | 18.737 | 21.485 | 20.805 |
| 34 | 3 | 27.867 | 21.726 | 20.057 | 18.640 | 20.251 | 20.327 |
| 35 | 3 | 26.812 | 21.884 | 20.337 | 18.927 | 19.951 | 20.802 |
| 36 | 3 | 27.769 | 23.538 | 22.289 | 18.625 | 22.272 | 20.196 |
| 37 | 3 | 27.112 | 22.976 | 21.395 | 19.008 | 20.529 | 20.770 |
| 38 | 3 | 28.789 | 23.704 | 22.228 | 18.946 | 21.795 | 20.610 |
| 39 | 3 | 27.281 | 22.843 | 21.455 | 18.941 | 21.160 | 20.785 |
| 40 | 3 | 28.021 | 23.366 | 22.418 | 18.979 | 22.517 | 20.783 |
| 41 | 3 | 26.478 | 21.900 | 20.741 | 19.257 | 21.546 | 20.668 |
| 42 | 3 | 27.350 | 22.980 | 21.699 | 18.770 | 22.199 | 20.715 |
| 43 | 3 | 28.498 | 23.730 | 22.469 | 18.633 | 22.989 | 20.560 |
| 44 | 3 | 26.137 | 22.987 | 21.236 | 19.243 | 21.084 | 20.869 |
| 45 | 3 | 27.593 | 23.773 | 22.448 | 18.946 | 22.487 | 20.686 |
| 46 | 3 | 27.205 | 23.077 | 21.969 | 18.463 | 22.296 | 20.872 |
| 47 | 3 | 27.702 | 22.640 | 21.496 | 18.821 | 21.277 | 21.307 |
| 48 | 3 | 25.498 | 22.660 | 21.433 | 18.977 | 21.524 | 20.682 |
| 49 | 3 | 26.852 | 23.159 | 22.302 | 19.317 | 22.703 | 21.316 |
| 50 | 4 | 26.464 | 22.511 | 21.046 | 18.848 | 22.122 | 21.020 |
| 51 | 4 | 27.747 | 22.732 | 21.530 | 19.550 | 22.467 | 21.333 |
| 52 | 4 | 27.629 | 22.617 | 20.503 | 19.319 | 21.330 | 20.418 |
| 53 | 4 | 26.647 | 24.153 | 22.669 | 19.205 | 23.426 | 21.026 |
| 54 | 4 | 26.395 | 22.931 | 21.230 | 18.919 | 22.267 | 21.316 |
| 55 | 4 | 28.592 | 22.706 | 21.220 | 19.541 | 21.882 | 21.689 |
| 56 | 4 | 26.627 | 22.918 | 21.630 | 19.020 | 22.065 | 21.372 |
| 57 | 4 | 26.763 | 22.599 | 20.918 | 19.657 | 21.563 | 20.559 |
| 58 | 4 | 27.127 | 23.293 | 21.998 | 19.310 | 21.903 | 21.277 |
| 59 | 4 | 28.283 | 24.663 | 23.146 | 19.588 | 23.790 | 20.545 |
| 60 | 4 | 27.153 | 23.983 | 22.567 | 19.188 | 22.812 | 21.280 |
| 61 | 4 | 28.149 | 25.123 | 24.158 | 19.642 | 24.406 | 21.228 |
| 63 | 4 | 29.631 | 26.162 | 25.040 | 19.828 | 25.471 | 21.788 |
| 64 | 4 | 29.080 | 24.465 | 23.276 | 19.230 | 23.584 | 20.838 |
| 65 | 4 | 28.311 | 23.477 | 22.002 | 18.938 | 22.714 | 20.751 |
| 67 | 4 | 27.830 | 23.911 | 22.654 | 18.906 | 22.815 | 20.552 |
| 68 | 5 | 26.292 | 23.541 | 21.957 | 18.671 | 21.900 | 20.522 |
| 69 | 5 | 28.253 | 24.065 | 22.582 | 19.364 | 23.527 | 20.812 |
| 70 | 5 | 28.477 | 24.274 | 23.135 | 19.091 | 22.911 | 20.651 |
| 71 | 5 | 28.911 | 24.762 | 23.531 | 19.588 | 23.077 | 20.778 |
| 72 | 5 | 26.108 | 24.169 | 22.255 | 19.523 | 22.197 | 21.082 |
| 73 | 5 | 26.785 | 23.454 | 22.461 | 18.724 | 23.382 | 20.814 |
| 74 | 5 | 26.868 | 22.669 | 21.181 | 18.767 | 20.968 | 20.917 |
| 75 | 5 | 27.579 | 23.116 | 21.691 | 18.599 | 22.547 | 20.258 |
| 76 | 5 | 26.740 | 23.656 | 21.992 | 19.340 | 21.731 | 20.610 |
| 77 | 5 | 27.392 | 23.425 | 22.162 | 18.991 | 21.627 | 20.536 |
| 78 | 5 | 28.042 | 25.289 | 24.182 | 19.540 | 24.377 | 21.159 |
| 79 | 5 | 28.421 | 23.932 | 22.269 | 19.927 | 21.440 | 21.359 |
| 80 | 5 | 28.314 | 23.850 | 22.205 | 19.514 | 21.941 | 20.894 |
| 81 | 5 | 26.565 | 22.940 | 21.673 | 18.398 | 21.895 | 20.500 |
| 82 | 5 | 27.776 | 23.484 | 21.888 | 19.192 | 22.054 | 20.667 |
| 85 | 5 | 28.075 | 24.540 | 23.426 | 20.036 | 23.664 | 20.738 |
| 86 | 6 | 27.271 | 23.380 | 21.811 | 18.824 | 21.952 | 20.524 |
| 87 | 6 | 27.964 | 24.668 | 22.968 | 19.612 | 22.805 | 21.488 |
| 88 | 6 | 27.970 | 24.398 | 22.865 | 19.792 | 22.889 | 21.197 |
| 89 | 6 | 29.853 | 25.039 | 23.680 | 19.405 | 22.315 | 20.973 |
| 90 | 6 | 28.102 | 24.542 | 22.923 | 19.829 | 21.856 | 21.196 |
| 91 | 6 | 28.910 | 23.126 | 21.388 | 19.485 | 20.362 | 20.884 |
| 92 | 6 | 28.402 | 22.852 | 21.473 | 19.378 | 21.829 | 21.293 |
| 93 | 6 | 29.644 | 24.348 | 22.985 | 20.206 | 23.462 | 21.701 |
| 94 | 6 | 28.509 | 24.893 | 23.373 | 19.351 | 22.399 | 20.736 |
| 95 | 6 | 29.957 | 24.207 | 22.951 | 20.365 | 22.441 | 21.833 |
| 96 | 6 | 29.497 | 24.447 | 23.137 | 19.304 | 22.593 | 20.696 |
| 97 | 6 | 29.620 | 24.992 | 23.147 | 20.345 | 21.382 | 21.720 |
| 98 | 6 | 29.208 | 24.620 | 22.911 | 20.144 | 21.527 | 21.642 |
| 99 | 6 | 28.986 | 23.928 | 22.543 | 19.370 | 21.901 | 20.923 |
| 100 | 6 | 27.997 | 23.739 | 22.492 | 19.626 | 21.872 | 21.217 |
| 101 | 6 | 28.353 | 24.638 | 23.379 | 19.317 | 23.889 | 20.730 |
| 102 | 7 | 28.205 | 24.200 | 22.883 | 18.833 | 23.669 | 21.120 |
| 103 | 7 | 27.196 | 24.934 | 23.415 | 20.481 | 24.011 | 21.671 |
| 104 | 7 | 30.953 | 24.330 | 23.057 | 19.322 | 22.846 | 20.801 |
| 105 | 7 | 28.824 | 24.710 | 22.773 | 18.974 | 22.774 | 20.957 |
| 106 | 7 | 27.489 | 24.678 | 22.969 | 19.683 | 22.907 | 21.790 |
| 107 | 7 | 28.865 | 25.903 | 24.735 | 19.554 | 25.114 | 21.375 |
| 108 | 7 | 27.251 | 24.817 | 23.556 | 19.528 | 24.815 | 21.540 |
| 109 | 7 | 28.240 | 24.848 | 23.482 | 19.772 | 22.788 | 21.526 |
| 110 | 7 | 29.818 | 24.888 | 23.425 | 19.492 | 23.018 | 21.611 |
| 111 | 7 | 28.800 | 25.098 | 23.693 | 19.241 | 24.289 | 21.033 |
| 112 | 7 | 28.708 | 25.056 | 23.686 | 19.309 | 24.231 | 21.160 |
| 113 | 7 | 28.633 | 24.003 | 22.148 | 19.774 | 21.485 | 21.370 |
| 114 | 7 | 27.922 | 22.110 | 20.230 | 18.951 | 20.269 | 21.330 |
| 115 | 7 | 26.588 | 24.214 | 22.772 | 18.804 | 22.858 | 20.904 |
| 116 | 7 | 26.647 | 24.860 | 23.478 | 19.566 | 22.936 | 21.456 |
| 117 | 7 | 27.979 | 23.844 | 22.176 | 19.390 | 21.631 | 21.370 |
| 118 | 8 | 27.519 | 23.154 | 21.395 | 19.162 | 20.720 | 21.320 |
| 119 | 8 | 26.847 | 23.652 | 22.166 | 18.720 | 22.261 | 20.634 |
| 120 | 8 | 27.182 | 24.981 | 23.578 | 18.939 | 23.643 | 20.668 |
| 121 | 8 | 28.690 | 25.004 | 23.635 | 18.595 | 25.017 | 20.893 |
| 122 | 8 | 26.791 | 22.970 | 21.372 | 18.536 | 21.010 | 20.839 |
| 123 | 8 | 27.635 | 23.669 | 22.354 | 19.454 | 22.145 | 21.259 |
| 124 | 8 | 26.813 | 24.780 | 23.608 | 19.030 | 24.751 | 20.781 |
| 125 | 8 | 27.794 | 23.531 | 22.247 | 18.831 | 22.643 | 20.298 |
| 126 | 8 | 27.437 | 23.517 | 21.944 | 19.141 | 22.149 | 20.582 |
| 127 | 8 | 27.672 | 24.377 | 23.186 | 19.512 | 23.516 | 21.295 |
| 128 | 8 | 28.946 | 25.167 | 23.760 | 18.804 | 23.399 | 20.501 |
| 129 | 8 | 27.220 | 23.668 | 22.002 | 18.806 | 21.820 | 20.762 |
| 130 | 8 | 28.292 | 24.434 | 22.986 | 18.389 | 23.112 | 20.157 |
| 131 | 8 | 26.428 | 24.310 | 23.437 | 19.344 | 24.241 | 21.214 |
| 133 | 8 | 26.050 | 25.380 | 22.988 | 19.503 | 23.451 | 21.542 |
| 134 | 8 | 26.822 | 23.547 | 22.251 | 18.637 | 22.196 | 20.451 |
| 135 | 9 | 27.871 | 24.331 | 23.232 | 19.939 | 23.316 | 21.323 |
| 136 | 9 | 27.646 | 24.793 | 23.775 | 19.598 | 24.715 | 20.851 |
| 137 | 9 | 27.301 | 24.439 | 22.932 | 19.704 | 22.944 | 21.153 |
| 140 | 9 | 28.017 | 24.455 | 23.213 | 19.273 | 23.779 | 20.906 |
| 141 | 9 | 27.142 | 23.889 | 22.427 | 19.815 | 22.619 | 20.846 |
| 142 | 9 | 26.512 | 23.141 | 21.916 | 19.158 | 22.465 | 20.808 |
| 144 | 9 | 27.157 | 25.293 | 24.308 | 19.720 | 25.198 | 20.585 |
| 145 | 9 | 26.937 | 24.130 | 23.091 | 19.793 | 23.482 | 21.230 |
| 146 | 9 | 27.358 | 24.289 | 23.249 | 19.141 | 24.115 | 20.491 |
| 148 | 9 | 27.298 | 22.331 | 20.979 | 18.928 | 21.316 | 20.835 |
| 149 | 9 | 27.978 | 24.622 | 23.222 | 19.427 | 23.479 | 20.706 |
| 151 | 9 | 28.645 | 24.458 | 22.675 | 19.291 | 22.895 | 20.634 |
| 153 | 9 | 27.530 | 23.188 | 21.826 | 19.245 | 22.510 | 21.019 |
| 154 | 9 | 27.466 | 23.499 | 22.362 | 19.428 | 22.828 | 20.791 |
| 156 | 9 | 27.552 | 24.845 | 23.666 | 19.702 | 24.223 | 21.130 |
| 158 | 9 | 27.371 | 22.077 | 20.369 | 19.499 | 20.273 | 20.713 |
| 165 | 10 | 27.494 | 23.593 | 22.017 | 19.388 | 21.549 | 20.881 |
| 167 | 10 | 25.556 | 22.783 | 21.153 | 19.338 | 21.158 | 20.298 |
| 174 | 10 | 27.759 | 23.629 | 22.238 | 19.321 | 22.422 | 21.309 |
| 175 | 10 | 26.406 | 23.822 | 21.998 | 19.095 | 21.959 | 21.123 |
| 176 | 10 | 28.278 | 24.682 | 23.294 | 19.667 | 24.456 | 21.546 |
| 177 | 10 | 27.309 | 23.200 | 21.791 | 18.738 | 22.419 | 19.945 |
| 179 | 10 | 27.593 | 23.323 | 21.958 | 19.594 | 21.903 | 20.649 |
| 180 | 10 | 25.883 | 22.323 | 21.183 | 19.331 | 22.052 | 20.655 |
| 181 | 10 | 27.495 | 23.856 | 22.732 | 19.238 | 22.982 | 20.763 |
| 182 | 10 | 29.587 | 25.894 | 23.827 | 21.004 | 21.975 | 23.123 |
| 183 | 10 | 26.260 | 21.777 | 19.792 | 18.556 | 19.917 | 21.101 |
| 184 | 10 | 26.295 | 22.542 | 20.795 | 18.770 | 20.607 | 20.624 |
| 186 | 10 | 27.320 | 23.282 | 21.802 | 18.690 | 21.581 | 20.670 |
| 187 | 10 | 27.953 | 22.823 | 21.839 | 18.619 | 22.310 | 20.404 |
| 188 | 10 | 26.630 | 22.914 | 21.565 | 19.147 | 21.660 | 20.782 |
| 189 | 10 | 26.256 | 21.870 | 19.810 | 19.101 | 19.496 | 20.595 |
| 190 | 10 | 25.515 | 21.982 | 20.090 | 18.611 | 20.274 | 20.559 |

^a^Mean raw cycle quantification (C_q_) values of triplicate PCR reactions are listed.

^b^Identification numbers of RNA samples of this study (S1 Table).

^c^All RT and PCR reactions for all RNA samples of a batch were performed together.
