# Supplementary material for: Whole blood microRNA expression may not be useful for screening non-small cell lung cancer
Source: PLoS One. 2017 Jul 25;12(7):e0181926. doi: 10.1371/journal.pone.0181926 (PMC5526508; doi:10.1371/journal.pone.0181926)
Supplement: S3 Table — (DOC) [file pone.0181926.s004.doc]

**S3 Table.** *Expression of microRNAs in the case and control cohorts with adjusted P <0.15 in one differential expression analysis.*

|  | *Mean (range; standard deviation)^a^* | |  |  |  |
| --- | --- | --- | --- | --- | --- |
| *MicroRNA* | *Cases (n=85)* | *Controls (n=76)* | *Fold-change^b^* | *P* | *Adjusted P^c^* |
| *miR-23b-3p* | 956 (467-2182; 294) | 795 (289-1498; 237) | 1.2 | 0.0002 | 0.1128 |
| *miR-363-5p* | 134 (71-247; 38) | 114 (50-194; 33) | 1.2 | 0.0004 | 0.1128 |
| *miR-1273g-3p* | 167 (100-300; 46) | 146 (61-220; 30) | 1.1 | 0.0020 | 0.1393 |
| *miR-197-3p* | 298 (160-611; 89) | 256 (87-467; 69) | 1.1 | 0.0012 | 0.1393 |
| *miR-219-5p* | 1345 (611-2258; 319) | 1191 (706-1841; 283) | 1.2 | 0.0034 | 0.1393 |
| *miR-3158-3p* | 58 (44-91; 8) | 54 (38-64; 5) | 1.0 | 0.0032 | 0.1393 |
| *miR-3613-3p* | 1932 (1178-2933; 378) | 1758 (1072-2492; 321) | 1.1 | 0.0023 | 0.1393 |
| *miR-4317* | 3724 (1513-6238; 1099) | 3205 (1152-6353; 1024) | 1.2 | 0.0011 | 0.1393 |
| *miR-4329* | 1715 (795-2938; 435) | 1967 (986-4302; 603) | 0.9 | 0.0033 | 0.1393 |
| *miR-4687-3p* | 75 (45-172; 20) | 68 (42-92; 10) | 1.1 | 0.0017 | 0.1393 |
| *miR-4708-3p* | 43 (32-68; 7) | 46 (35-60; 6) | 0.9 | 0.0026 | 0.1393 |
| *miR-4769-3p* | 68 (47-109; 11) | 63 (40-98; 11) | 1.0 | 0.0028 | 0.1393 |
| *miR-542-3p* | 75 (52-111; 13) | 82 (50-130; 16) | 0.9 | 0.0035 | 0.1393 |
| *miR-551a* | 60 (53-67; 3) | 59 (48-66; 3) | 1.0 | 0.0036 | 0.1393 |
| *miR-5684* | 54 (45-85; 6) | 51 (40-69; 5) | 1.0 | 0.0015 | 0.1393 |
| *miR-129-1-3p* | 144 (85-340; 52) | 124 (64-200; 29) | 1.1 | 0.0043 | 0.1400 |
| *miR-194-5p* | 423 (154-867; 133) | 368 (118-734; 106) | 1.1 | 0.0043 | 0.1400 |
| *miR-27a-3p* | 58 (41-80; 6) | 55 (38-73; 7) | 1.1 | 0.0039 | 0.1400 |
| *miR-4787-5p* | 1453 (804-2649; 379) | 1654 (817-3877; 506) | 0.9 | 0.0046 | 0.1421 |

^a^Microarray signal values.

^b^Ratio of median values of microarray signals of cases and controls.

^c^P value adjusted with the Benjamini-Hochberg method for a maximum false discovery rate of 5%.
