# Supplementary material for: Whole blood microRNA expression may not be useful for screening non-small cell lung cancer
Source: PLoS One. 2017 Jul 25;12(7):e0181926. doi: 10.1371/journal.pone.0181926 (PMC5526508; doi:10.1371/journal.pone.0181926)
Supplement: S1 Text — (DOC) [file pone.0181926.s005.doc]

**S1 Text.** *R code used for estimating power of study.*

# Environment: Mac OS X 10.6.8; R 2.14.1; Biobase 2.14.0; limma 3.10.3; SSPA 1.12.0

library('Biobase')

library('limma')

library('SSPA')

# Pilot data; normalized Hy3 signal values (not log transformed) for the 395 'expressed' human microRNAs (387 microarray probes) of Patnaik et al., PLOS ONE, 2012

exp <- readExpressionSet(exprsFile='hy3ExpHsaMir.txt', sep='\t', row.names=1, header=T)

# Cohort membership; c for cancer case, h for control

classes <- factor(c(rep('c', 23), rep('h', 22)))

# Obtain moderated t statistics in differential expression analysis using limma packagae

design <- model.matrix(~0 + classes)

colnames(design) <- levels(classes)

contrast <- makeContrasts(c-h, levels=design)

fit <- lmFit(exp, design)

fit <- contrasts.fit(fit, contrast)

fitModerated <- eBayes(fit)

nu <- fit$df.residual[1]

nu0 <- fitModerated$df.prior

pd <- pilotData(name='exp', testStatistics=fitModerated$t[,1], sampleSizeA=22 + nu0/2, sampleSizeB=23 + nu0/2, dof=nu + nu0, nullDist='student')

# The sampleSize function performs the estimation of the proportion of non-differentially expressed genes; the 'Langaas' method is used by default. Gives warning 'Resolution should be smaller than number of test statistics'

ss <- sampleSize(pd)

# Plot effect-size density

plotEffectSize(ss, type='l', lwd=3, sub=NULL)

# Plot power estimates; gives "'full precision may not have been achieved in 'pnt{final}'" warnings

samplesizes = seq(5, 120, by=5)

pwr <- Power(ss, threshold=0.2, plot=FALSE, samplesizes=samplesizes + nu0/2, fdr=c(0.05, 0.1, 0.2))

matplot(samplesizes, pwr, ylim=c(0, 1), type='b', col=c(1:3), lty=1, pch=20, lwd=3, ylab='Power', xlab='Sample-size/group', main='Power curves')

legend('bottomright', colnames(pwr), col=c(1:3), lty=1)

# Gives 0.942, 0.974 and 0.999 power at a=0.05, 0.1 and 0.2, resp., at sample size=75

pwr
