# Supplementary material for: Whole blood microRNA expression may not be useful for screening non-small cell lung cancer
Source: PLoS One. 2017 Jul 25;12(7):e0181926. doi: 10.1371/journal.pone.0181926 (PMC5526508; doi:10.1371/journal.pone.0181926)
Supplement: S2 Text — (DOC) [file pone.0181926.s006.doc]

**S2 Text.** *R code used for processing of raw microarray data.*

# Environment: Mac OS X 10.6.8; R 2.14.1; Biobase 2.14.0; futile.matrix 1.1.2; limma 3.10.3; reshape2 1.2.1

library('Biobase')

library('futile.matrix')

library('limma')

library('reshape2')

# File sampleinfo.txt has sample and data information for all arrays of this study in format required by limma

targets <- readTargets('sampleinfo.txt')

files <- targets[, c('Cy3', 'Cy5')]

# Read data with flag-values, assigning a value of 0 if the Imagene flag-value is >0, and a 1 if <1; the array layout file (.gal file) for the microarray platform is obtained from Exiqon® website

myWtFun <- function(exclude.flags=c(1, 2, 3, 4, 5, 6, 7)) function(obj) 1-(obj$Flag %in% exclude.flags)

RG <- read.maimages(files, source='imagene', wt.fun=myWtFun(c(1, 2, 3, 4, 5, 6, 7)))

colnames(RG) <- targets$Name

RG$genes <- readGAL('gal_208500,208501,208502,208510_lot35004-35004_hsa-and-related-vira_from_mb180,miRPlus.gal')

RG$printer <- getLayout(RG$genes)

# Background-correction using 'normexp' method with offset=10

RG <- backgroundCorrect(RG, method='normexp', offset=10)

# Global 'loess' within-aray normalization with span=1/3

MA <- normalizeWithinArrays(RG, weights=RG$weights, method='loess', span=1/3)

# Remove arrays with data of poor quality (RNA sample IDs 3, 168, 178 and 191)

MA <- MA[,-c(3, 157, 167, 179)]

# 'Quantile' between-array normalization

MA <- normalizeBetweenArrays(MA, method='quantile')

RG <- new('RGList', RG.MA(MA))

# Probe-set summarization using mean if max/min <cutoffAgg; else, median

cutoffAgg <- 1.5

myAggFun <- function(x){ifelse(cutoffAgg<(max(x)/min(x)), median(x), mean(x))}

Hy3 <- RG$G

Hy5 <- RG$R

FC <- Hy3/Hy5

l<-list('Hy3','Hy5', 'FC')

for(i in 1:length(l)){

dat <- get(l[[i]])

rownames(dat) <- RG$genes$Name

dat <- dcast(melt(dat), Var1~Var2, myAggFun, fill=NaN)

rownames(dat) <- dat$Var1

dat$Var1 <- NULL

assign(l[[i]], dat)

}

# Filter for 'expressed' RNA; retain if more than a cutoffExpFrac of samples have Hy3 values >cutoffExpFold-times summarized 'Empty' spot value; values used here are same as those used for Patnaik et al., PLOS ONE, 2012

cutoffExpFold <- 3

cutoffExpFrac <- 0.25

hy3RelEmpty <- apply(scale(Hy3, center=FALSE, scale=t(Hy3[c('Empty'),])), 1:2, function(x){ifelse(x>cutoffExpFold, 1, 0)})

rnaExp <- rownames(hy3RelEmpty[rowSums(hy3RelEmpty) > ncol(hy3RelEmpty)*cutoffExpFrac,])

# Filter for human RNAs

patHsaMir <- c('(hsa-((miR)|(let)))')

# Save Hy3, Hy5 and Hy3/Hy5 ratio values for 'expressed' human microRNA expression data-set

write.table(select(Hy3[rnaExp,], row.pat=patHsaMir), file='hy3ExpHsaMir.txt', sep="\t", col.names=NA)

write.table(select(Hy5[rnaExp,], row.pat=patHsaMir), file='hy5ExpHsaMir.txt', sep="\t", col.names=NA)

write.table(select(FC[rnaExp,], row.pat=patHsaMir), file='FCExpHsaMir.txt', sep="\t", col.names=NA)
