# Supplementary material for: Whole blood microRNA expression may not be useful for screening non-small cell lung cancer
Source: PLoS One. 2017 Jul 25;12(7):e0181926. doi: 10.1371/journal.pone.0181926 (PMC5526508; doi:10.1371/journal.pone.0181926)
Supplement: S3 Text — (DOC) [file pone.0181926.s007.doc]

**S3 Text.** *R code used for one set of classification analyses.*

These analyses examine discrimination of cases and controls by their microRNA profiles using linear kernel support vector machines (SVM) and top-scoring pair (TSP) methods, and leave-one-out (LOOCV) and Monte Carlo (MCCV) cross-validations are performed using CMA and tspair Bioconductor packages. Expression dataset for 85 cases and 76 controls for 598 microRNAs identified as expressed in the study is analyzed. MCCV uses 131 samples for training, and 1,000 iterations. In case of SVM, classifiers are tuned with a value set of 0.1, 0.2, 0.5, 1, 2, 5, 10, 20, 50 for *cost*, and consist of 15 differentially expressed microRNAs (genes) as identified by the limma method. Outputs are in-terminal, and include classification method performance characteristics such as accuracy and sensitivity, and numbers and names of genes that appear in the classifiers. The code has separate sections for each type of classifier and cross-validation method.

# Environment: Mac OS X 10.6.8, R 2.14.1, CMA 1.12.0, tspair 1.12.0, WilcoxCV 1.0.2

library('CMA')

library('tspair')

library('WilcoxCV')

# Input data has normalized Hy3 signal values (not log-transformed) for the 598 'expressed' human microRNAs

exp <- as.matrix(read.table(file='hy3ExpHsaMir.txt', sep='\t', row.names=1, header=T, check.names=F))

# Sample IDs for the 85 cases and 76 controls; for subjects with replicate samples, using the replicate with a smaller ID value

cases <- c('1', '2', '4', '5', '6', '7', '8', '9', '10', '21', '22', '23', '24', '25', '26', '27', '28', '29', '30', '41', '42', '43', '44', '45', '46', '47', '48', '49', '50', '61', '63', '64', '65', '67', '68', '69', '70', '81', '82', '85', '86', '87', '88', '89', '90', '101', '102', '103', '104', '105', '106', '107', '108', '109', '110', '111', '112', '121', '122', '123', '124', '125', '126', '127', '149', '151', '153', '154', '156', '158', '165', '167', '174', '175', '176', '177', '179', '180', '181', '183', '184', '186', '187', '188', '189')

controls <- c('11', '12', '13', '14', '15', '16', '17', '18', '19', '20', '31', '32', '33', '34', '35', '36', '37', '38', '39', '40', '51', '52', '53', '54', '55', '56', '57', '58', '59', '60', '71', '72', '73', '74', '75', '76', '77', '78', '79', '80', '91', '92', '93', '94', '95', '96', '97', '98', '99', '100', '113', '114', '115', '116', '117', '118', '119', '120', '128', '129', '130', '131', '133', '134', '135', '136', '137', '140', '141', '142', '144', '145', '146', '148', '182', '190')

classes <- factor(c(rep(0, length(cases)), rep(1, length(controls))))

# Class label for 'positive' items in 'classes'

yesPredVal = 1

exp <- exp[,c(cases, controls)]

## TSP - LOOCV

res <- matrix(nrow=0, ncol=4)

colnames(res) <- c('iteration', 'score', 'gene1', 'gene2')

tp <- tn <- fp <- fn <- 0

out = 'tspair LOOCV:\nSample\tClass label\tPredicted class label\n'

for(i in 1:ncol(exp)){

# Actual prediction work

tsp <- tspcalc(exp[, -i], classes[-i])

pred <- predict(tsp, exp)[i]

known <- classes[i]

if(pred == known){

if(pred == yesPredVal){tp <- tp+1}

else{tn <- tn+1}

}else{

if(pred == yesPredVal){fp <- fp+1}

else{fn <- fn+1}

}

out = paste(out, colnames(exp)[i], '\t', known, '\t', pred, '\n', sep='')

res <- rbind(res, c(i, tsp$tspscore[1], rownames(exp)[tsp$index[1, 1]], rownames(exp)[tsp$index[1, 2]]))

}

# Output

out = paste(out, ncol(exp), ' predictions; accuracy, sensitivity, specificity, PPV and NPV in fractions are ', round((tp+tn)/(tp+tn+fp+fn), 3), ', ', round(tp/(tp+fn), 3) , ', ', round(tn/(tn+fp), 3), ', ', round(tp/(tp+fp), 3) , ', and ', round(tn/(tn+fn), 3), ', respectively; mean (range; SD) of TSP scores for the topmost TS pairs is ', round(mean(as.numeric(res[, 'score'])), 2), ' (', round(min(as.numeric(res[, 'score'])), 2), '-', round(max(as.numeric(res[, 'score'])), 2), '; ', round(sd(as.numeric(res[, 'score'])), 2), '). There are a total of ', length(unique(res[, 'gene1'])), ' and ', length(unique(res[, 'gene2'])), ' unique genes for gene-1 and -2, respectively, in ', ncol(exp), ' topmost TS pairs with following occurrence frequencies in fractions:\n', sep='')

tspGeneTab <- as.matrix(append(res[, 'gene1'], res[, 'gene2']))

tspUnqGene <- unique(tspGeneTab)

for(i in 1:length(tspUnqGene)){

out = paste(out, tspUnqGene[i], round(length(tspGeneTab[tspGeneTab[, 1]==tspUnqGene[i], ])/ncol(exp), 3), '\n', sep='\t')

}

cat(out)

## SVM - LOOCV

diffTest = 'limma'

diffGeneNum = 15

svmCost <- c(0.1, 0.2, 0.5, 1, 2, 5, 10, 20, 50)

tp <- tn <- fp <- fn <- 0

# Actual prediction work

lsets <- GenerateLearningsets(n=ncol(exp), y=classes, method=c('LOOCV'))

svm <- classification(t(exp), factor(classes), learningsets=lsets, genesellist=list(method=diffTest), classifier=svmCMA, nbgene= diffGeneNum, tuninglist=list(grids=list(cost=svmCost)), probability=T, models=T)

svm <- join(svm)

# Output. Note that CMA internally re-names class labels.

out = paste('SVM LOOCV with ', diffTest, ' and nbgene=', diffGeneNum, ':\nSample\tClass label\tClass label as per CMA\tPredicted class label as per CMA\n', sep='')

for(i in 1:ncol(exp)){

# since this is the way LOOCV training sets are generated in CMA

j <- ncol(exp)-(i-1)

pred <- svm@yhat[j]

known <- svm@y[j]

if(pred == known){

if(pred == yesPredVal){tp <- tp+1}

else{tn <- tn+1}

}else{

if(pred == yesPredVal){fp <- fp+1}

else{fn <- fn+1}

}

out = paste(out, colnames(exp)[i], '\t', classes[i], '\t', known, '\t', pred, '\n', sep='')

}

out = paste(out, ncol(exp), ' predictions; accuracy, sensitivity, specificity, PPV and NPV in fractions are ', round((tp+tn)/(tp+tn+fp+fn), 3), ', ', round(tp/(tp+fn), 3) , ', ', round(tn/(tn+fp), 3), ', ', round(tp/(tp+fp), 3) , ', and ', round(tn/(tn+fn), 3), ', respectively. ', sep='')

svmGenes <- GeneSelection(t(exp), classes, learningsets=lsets, method=diffTest)

temp <- numeric()

for(i in 1:ncol(exp)){

temp <- c(temp, toplist(svmGenes, k=diffGeneNum, iter=i, show=F)$index)

}

temp2 <- unique(temp)

out = paste(out, ' A total of ', length(temp2), ' genes occur in the ', ncol(exp), ' classifiers, with occurrence frequencies in fractions of:\n', sep='')

for(i in 1:length(temp2)){

out = paste(out, rownames(exp)[temp2[i]], '\t', round(sum(temp==temp2[i])/ncol(exp), 3), '\n', sep='')

}

cat(out);

## TSP - MCCV

myNumFun <- function(x, y){round(y(as.numeric(x), na.rm=T), 4)}

set.seed(631)

out = ''

niter = 1000

ntest = 30

result <- matrix(nrow=0, ncol=10)

colnames(result) <- c('trainSetSize', 'iteration', 'acc', 'sens', 'spec', 'ppv', 'npv', 'score', 'gene1', 'gene2')

diffGenesAll <- c()

# Generate 2*niter training/test sets but use niter sets (valid TSP calc. requires min. 2 classes)

set <- generate.split(niter=2*niter, n=ncol(exp), ntest=ntest)

actualIters = 0

for(h in 1:(2*niter)){

if(actualIters > niter-1){

break;

}

# Actual prediction work

trExp <- exp[, -set[h, ]]

tsExp <- exp[, set[h, ]]

trClasses <- classes[-set[h, ]]

tsClasses <- classes[set[h, ]]

if(1 < length(unique(trClasses))){

actualIters = actualIters+1

tsp <- tspcalc(trExp, trClasses)

tp <- tn <- fp <- fn <- 0

for(i in 1:ncol(tsExp)){

pred <- predict(tsp, tsExp)[i]

known <- tsClasses[i]

if(pred == known){

if(pred == yesPredVal){tp <- tp+1}

else{tn <- tn+1}

}else{

if(pred == yesPredVal){fp <- fp+1}

else{fn <- fn+1}

}

}

result <- rbind(result, c(ncol(exp)-ntest, h, (tp+tn)/(tp+tn+fp+fn), tp/(tp+fn), tn/(tn+fp), tp/(tp+fp), tn/(tn+fn), tsp$tspscore[1], rownames(exp)[tsp$index[1, 1]], rownames(exp)[tsp$index[1, 2]]))

diffGenesAll <- c(diffGenesAll, rownames(exp)[tsp$index[1, 1]], rownames(exp)[tsp$index[1, 2]])

} # end if valid TSP

} # end for h

# Output

out = paste(out, '\nTSP MCCV using ', niter, ' attempted iterations and ', actualIters, ' successful iterations, with ', ncol(exp)-ntest, ' of ', ncol(exp), ' total samples used for training:\nThe means (ranges; SDs) of prediction accuracy, sensitivity, specificity, PPV and NPV in fractions are ', myNumFun(result[, 'acc'], mean), ' (', myNumFun(result[, 'acc'], min), '-', myNumFun(result[, 'acc'], max), '; ', myNumFun(result[, 'acc'], sd), '), ', myNumFun(result[, 'sens'], mean), ' (', myNumFun(result[, 'sens'], min), '-', myNumFun(result[, 'sens'], max), '; ', myNumFun(result[, 'sens'], sd), '), ', myNumFun(result[, 'spec'], mean), ' (', myNumFun(result[, 'spec'], min), '-', myNumFun(result[, 'spec'], max), '; ', myNumFun(result[, 'spec'], sd), '), ', myNumFun(result[, 'ppv'], mean), ' (', myNumFun(result[, 'ppv'], min), '-', myNumFun(result[, 'ppv'], max), '; ', myNumFun(result[, 'ppv'], sd), '), and ', myNumFun(result[, 'npv'], mean), ' (', myNumFun(result[, 'npv'], min), '-', myNumFun(result[, 'npv'], max), '; ', myNumFun(result[, 'npv'], sd), '), respectively. The mean (range; SD) of the topmost TSP scores is ', myNumFun(result[, 'score'], mean), ' (', myNumFun(result[, 'score'], min), '-', myNumFun(result[, 'score'], max), '; ', myNumFun(result[, 'score'], sd), '). There are a total of ', length(unique(result['gene1'])), ' and ', length(unique(result['gene2'])), ' unique genes for gene-1 and -2, respectively, in the ', actualIters, ' topmost TS pairs with following occurrence frequencies, in fractions:\n', sep='')

diffGenesAllUnq <- unique(diffGenesAll)

for(i in 1:length(diffGenesAllUnq)){

out = paste(out, diffGenesAllUnq[i], round(sum(diffGenesAll == diffGenesAllUnq[i])/actualIters, 4), '\n', sep='\t')

}

cat(out)

## SVM - MCCV

diffTest = 'limma'

diffGeneNum = 15

svmCost <- c(0.1, 0.2, 0.5, 1, 2, 5, 10, 20, 50)

result <- matrix(nrow=0, ncol=7)

colnames(result) <- c('trainSetSize', 'iteration', 'acc', 'sens', 'spec', 'ppv', 'npv')

diffGenes <- numeric()

# Generate learning-sets with 2*niter members in case some have to be removed; valid SVM classification requires min. 2 classes; further, inner loop validation requires certain number of members of each class in each training set

lsets <- GenerateLearningsets(n=ncol(exp), y=classes, method=c('MCCV'), niter=2*niter, ntrain=ncol(exp)-ntest)

temp <- lsets@learnmatrix

for(i in 1:(2*niter)){

if(2 > length(unique(classes[lsets@learnmatrix[i, ]]))){

temp <- lsets@learnmatrix[-i, ]

}

}

lsets@learnmatrix <- temp[1:niter, ]

lsets@iter <- niter

# Genes in classifiers

svmGenes <- GeneSelection(t(exp), classes, learningsets=lsets, method=diffTest)

svmTune <- tune(t(exp), factor(classes), learningsets=lsets, genesel=svmGenes, classifier=svmCMA, nbgene=diffGeneNum, grids=list(cost=svmCost))

# Actual prediction work

svm <- classification(t(exp), factor(classes), learningsets=lsets, genesel=svmGenes, classifier=svmCMA, nbgene=diffGeneNum, tuneres=svmTune, probability=T, models=T)

svm <- join(svm)

actualIters=0

for(h in 1:niter){

m <- ntest*(h-1)

if(1 < length(unique(classes[-lsets@learnmatrix[h,]]))){

actualIters = actualIters+1

tp <- tn <- fp <- fn <- 0

for(i in 1:ntest){

pred <- svm@yhat[m+i]

known <- svm@y[m+i]

if(pred == known){

if(pred == yesPredVal){tp <- tp+1}

else{tn <- tn+1}

}else{

if(pred == yesPredVal){fp <- fp+1}

else{fn <- fn+1}

}

}

result <- rbind(result, c(ncol(exp)-ntest, h, (tp+tn)/(tp+tn+fp+fn), tp/(tp+fn), tn/(tn+fp), tp/(tp+fp), tn/(tn+fn)))

diffGenes <- c(diffGenes, toplist(svmGenes, k=diffGeneNum, iter=h, show=F)$index)

} # end if valid SVM

} # end for h

# Output performance characteristics

out = paste(out, 'SVM MCCV using ', niter, ' attempted iterations and ', actualIters, ' successful iterations, with ', ncol(exp)-ntest, ' of ', ncol(exp), ' total samples used for training:\nThe means (ranges; SDs) of prediction accuracy, sensitivity, specificity, PPV and NPV in fractions are ', myNumFun(result[, 'acc'],mean), ' (', myNumFun(result[, 'acc'], min), '-', myNumFun(result[, 'acc'], max), '; ', myNumFun(result[, 'acc'], sd), '), ', myNumFun(result[, 'sens'], mean), ' (', myNumFun(result[, 'sens'], min), '-', myNumFun(result[, 'sens'], max), '; ', myNumFun(result[, 'sens'], sd), '), ', myNumFun(result[, 'spec'], mean), ' (', myNumFun(result[, 'spec'], min), '-', myNumFun(result[, 'spec'], max), '; ', myNumFun(result[, 'spec'], sd), '), ', myNumFun(result[, 'ppv'], mean), ' (', myNumFun(result[, 'ppv'], min), '-', myNumFun(result[, 'ppv'], max), '; ', myNumFun(result[, 'ppv'], sd), '), and ', myNumFun(result[, 'npv'], mean), ' (', myNumFun(result[, 'npv'], min), '-', myNumFun(result[, 'npv'], max), '; ', myNumFun(result[, 'npv'], sd), '), respectively.\n', sep='')

# Output classifier genes

diffGenesUnq <- unique(diffGenes)

out = paste(out, 'A total of ', length(diffGenesUnq), ' genes occur in the ', actualIters, ' classifiers, with occurrence frequencies in fractions:\n', sep='')

for(i in 1:length(diffGenesUnq)){

out = paste(out, rownames(exp)[diffGenesUnq[i]], '\t', round(sum(diffGenes == diffGenesUnq[i])/actualIters, 3), '\n', sep='')

}

cat(out)
